# Transcriptional Landscape and Regulatory Roles of Small Noncoding RNAs in the Oxidative Stress Response of the Haloarchaeon Haloferax volcanii

**DOI:** 10.1128/JB.00779-17

**Published:** 2018-04-09

**Authors:** Diego Rivera Gelsinger, Jocelyne DiRuggiero

**Affiliations:** a Department of Biology, The Johns Hopkins University, Baltimore, Maryland, USA; University of Illinois at Urbana Champaign

**Keywords:** archaea, extreme environments, noncoding RNA, oxidative stress, small RNA, transcriptional regulation

## Abstract

Haloarchaea in their natural environment are exposed to hypersalinity, intense solar radiation, and desiccation, all of which generate high levels of oxidative stress. Previous work has shown that haloarchaea are an order of magnitude more resistant to oxidative stress than most mesophilic organisms. Despite this resistance, the pathways haloarchaea use to respond to oxidative stress damage are similar to those of nonresistant organisms, suggesting that regulatory processes might be key to their robustness. Recently, small regulatory noncoding RNAs (sRNAs) were discovered in Archaea under a variety of environmental conditions. We report here the transcriptional landscape and functional roles of sRNAs in the regulation of the oxidative stress response of the model haloarchaeon Haloferax volcanii. Thousands of sRNAs, both intergenic and antisense, were discovered using strand-specific sRNA sequencing (sRNA-seq), comprising 25 to 30% of the total transcriptome under no-challenge and oxidative stress conditions, respectively. We identified hundreds of differentially expressed sRNAs in response to hydrogen peroxide-induced oxidative stress in H. volcanii. The targets of a group of antisense sRNAs decreased in expression when these sRNAs were upregulated, suggesting that sRNAs are potentially playing a negative regulatory role on mRNA targets at the transcript level. Target enrichment of these antisense sRNAs included mRNAs involved in transposon mobility, chemotaxis signaling, peptidase activity, and transcription factors.

**IMPORTANCE** While a substantial body of experimental work has been done to uncover the functions of small regulatory noncoding RNAs (sRNAs) in gene regulation in Bacteria and Eukarya, the functional roles of sRNAs in Archaea are still poorly understood. This study is the first to establish the regulatory effects of sRNAs on mRNAs during the oxidative stress response in the haloarchaeon Haloferax volcanii. Our work demonstrates that common principles for the response to a major cellular stress exist across the 3 domains of life while uncovering pathways that might be specific to the Archaea. This work also underscores the relevance of sRNAs in adaptation to extreme environmental conditions.

## INTRODUCTION

Microbial communities that reside inside halite nodules from Salars in the Atacama Desert, Chile, are under extreme environmental pressures due to hypersalinity, intense solar radiation, and frequent desiccation-hydration cycles, which all generate high levels of oxidative stress (1, 2). Oxidative stress occurs when the level of reactive oxygen species (ROS) produced in cells overwhelms antioxidant defense mechanisms and damage accumulates (3). Through metagenomic studies, we found the dominant populations in these salt rocks to be haloarchaea such as Haloferax and Halobacterium (4). These halophilic microorganisms are members of the third domain of life, the Archaea. Haloarchaea have previously been shown to be highly resistant to ROS damage, withstanding many times what Escherichia coli and other radiation-sensitive organisms can survive (5–7). The haloarchaeon Halobacterium salinarum has been shown to use a wide range of strategies to combat damage from oxidative stress, including multiple copies of genomes (polyploidy) as the substrate for DNA repair, functional redundancy of DNA repair and detoxification enzymes (e.g., catalase), increased cytosolic manganese complexes to scavenge ROS, and differential regulation of genes in response to stress (5–9). However, pathways for DNA repair and protein turnover in haloarchaea are nearly identical to those in nonresistant bacteria and eukarya, suggesting that the regulation of these processes in response to oxidative stress might be key to their robustness. Previous work with H. salinarum oxidative stress gene regulatory networks revealed that a single transcription factor, RosR, regulates the appropriate dynamic response of nearly 300 genes to reactive oxygen species stress (5). This work demonstrated that the oxidative stress response in H. salinarum impacted a wide array of cellular processes, engaging at least 50% of all the genes (2). These results underline the importance of gene regulation in haloarchaea for responding to and counteracting the damage caused by oxidative stress.

In addition to transcription factors, small regulatory RNAs (sRNAs) similarly act as global gene regulators (10). Small RNAs (sRNAs) are ubiquitously found in Bacteria and Eukarya, playing large-scale roles in gene regulation, transposable element silencing, defense against disease state, and foreign elements (11–14). Several types of sRNAs have been identified in the Eukarya (microRNAs [miRNAs], small interfering RNAs [siRNAs], and Piwi-interacting RNAs [piRNAs]) and they are typically 20 to 25 nucleotides (nt) long. Their major mode of interaction is through base pairing to the 3′ untranslated region (UTR) of their target mRNAs, inhibiting translation or triggering target degradation with associated protein components (Argonautes) (10). Bacterial sRNAs have been shown to modulate core metabolic functions and stress-related responses, such as nutrient deprivation, by binding target mRNAs and causing their degradation or preventing translation (11, 15). Most of the functionally characterized sRNAs in Bacteria bind the 5′ UTR of their target mRNA and are longer than their eukaryal counterparts, with sizes ranging from 50 to 500 nt. These sRNAs can target multiple genes, including key transcription factors and regulators (11, 15, 16). As a consequence, a single sRNA can modulate the expression of large regulons and thus have a significant effect on metabolic processes. For example, the bacterial sRNA OxyS, which is dramatically induced by oxidative stress, regulates the expression of approximately 40 genes and interacts directly with eight target mRNAs (11).

sRNAs have been discovered to be abundant in Archaea, more specifically in haloarchaea, in response to a variety of environmental conditions, but the functional roles of these RNAs still remain poorly understood and a link between sRNAs expression and oxidative stress response has not been established (13, 17–24). Only a few studies on sRNAs in hyperthermophiles, methanogens, and the haloarchaeon Haloferax volcanii have been reported so far (13, 17–24). In H. volcanii, large numbers of intergenic and antisense-encoded sRNAs, 145 and 45, respectively, were discovered by using microarray in addition to a novel class of sRNAs recently described in eukaryotes, tRNA-derived fragments (tRFs), and a new study found thousands of sRNAs present in this organism (19, 24, 25). In Sulfolobus solfataricus, 125 *trans*-encoded sRNAs and 185 *cis*-antisense sRNAs were identified using high-throughput sequencing (HTS), suggesting that 6.1% of all genes in S. solfataricus are associated with sRNAs (26). A comparative genome analysis of Methanosarcina mazei, M. barkeri, and M. acetivorans revealed that 30% of the antisense and 21% of the intergenic sRNAs identified were conserved across the 3 species (27). Coimmunoprecipitation with the Lsm protein (archaeal Hfq homolog) was used to “capture” sRNAs (17), but its functional role remains to be elucidated. While Ago homologs have also been found in archaeal genomes, there is no evidence for eukaryote-like RNA interference in these organisms (28). Rather, a defensive role against foreign genetic material was recently proposed, whereby archaeal Ago proteins direct guide-dependent cleavage of foreign DNA (29–31). Altogether, these studies suggest that sRNAs are as widespread and abundant in the Archaea as in the Bacteria and Eukarya.

Target mRNA identification of sRNAs has proven to be difficult within the Archaea but is a necessary task for uncovering sRNA functionality. Transcriptome sequencing (RNA-seq) in M. mazei cultures, grown under nitrogen starvation conditions, showed the differential expression of a number of sRNAs in response to nitrogen availability and enabled the identification of the first *in vivo* target for archaeal intergenic sRNAs (27, 32). The potential target for sRNA_162_ is a bicistronic mRNA encoding a transcription factor involved in regulating the switch between carbon sources and a protein of unknown function (32). In Pyrobaculum, 3 antisense sRNAs were found opposite a ferric uptake regulator, a triose-phosphate isomerase, and transcription factor B, supporting a potential role for archaeal antisense sRNA in the regulation of iron, transcription, and core metabolism (33). sRNA deletion mutants can be used to identify potential biological functions and target genes. Deletion strains were successfully generated for H. volcanii, and phenotyping of the sRNAs deletion mutants revealed several severe growth defects under high temperatures, low salt concentrations, or specific carbon sources (9, 22). While these studies revealed that sRNAs likely play essential roles in the physiological responses to environmental challenges in the Archaea, the functional roles and mechanisms of action of these important posttranscriptional regulators still remain unknown. Furthermore, no work has been done to investigate archaeal sRNAs in response to oxidative stress, a universal and frequent stressor in all domains of life that results in extensive cellular damage. To determine the impact of sRNAs during the oxidative stress response, we assessed the H. volcanii transcriptional landscape under no-challenge and oxidative stress conditions using comparative strand-specific small RNA sequencing (sRNA-seq).

## RESULTS

To identify globally small noncoding RNAs differentially expressed in response to oxidative stress in H. volcanii, we exposed 5 replicate cultures of H. volcanii to 2 mM H_2_O_2_, a dose that resulted in the survival of 80% of the cells (see Fig. S1 in the supplemental material). RNA from these H_2_O_2_-treated cultures and from no-challenge cultures (controls) was sequenced using a strand-specific size-selected (50 to 500 nt) sRNA library preparation essential for sRNA discovery.

### Small noncoding RNA discovery in H. volcanii and normalized expression values.

**FIG 1:**
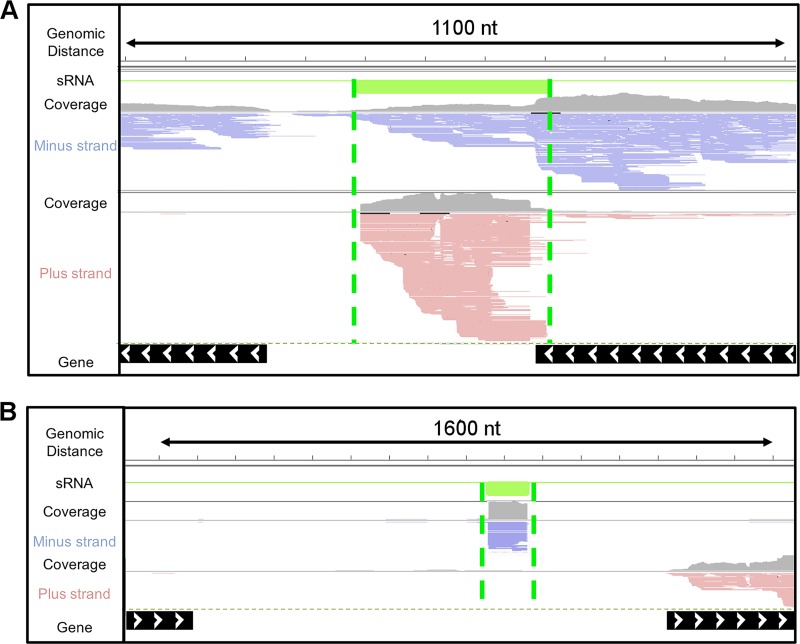
Genome viewer of antisense sRNAs (*cis* acting) (A) and intergenic sRNAs (*trans* acting) (B). Paired-end reads (100 bases) were mapped to the H. volcanii NCBI reference genome. Reference genes are marked as black lines with arrowheads indicating their location on the plus strand (>) or minus strand (<). Reads marked in red are transcribed from the minus strand, while blue reads are transcribed from the plus strand. Untranslated regions were predicted using Rockhopper2 (pink lines). Green lines mark discovered sRNAs. Coverage plots are in gray.

**FIG 2:**
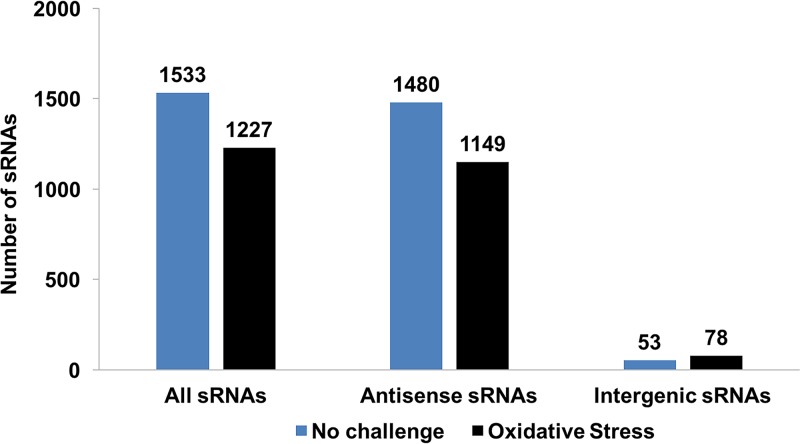
Number of sRNAs (total, antisense, and intergenic) discovered under no-challenge and H_2_O_2_ challenge conditions.

We obtained at total of 137 million sequence reads (41 Gb) across all replicates and conditions. Following quality control and reference-based read mapping, we intersected the mapped reads against the H. volcanii reference genome to discover sRNA transcripts that we classified as antisense (overlapping a gene and/or its regulatory elements on the opposite strand) (Fig. 1A) and intergenic (in a noncoding region between two genes) (Fig. 1B). We were unable to identify previously described *cis*-internal sRNAs (24), because with our RNA-seq strategies, those transcripts would be confounded with the corresponding gene transcripts. To further validate the sRNAs we identified and reduce transcriptional noise in our data, we applied a rigorous two-pronged *in silico* approach. First, we used a stringent threshold requiring an sRNA (i) to be present in at least four of five biological replicate libraries, and (ii) to have a minimum expression of 40 transcripts per million ([TPM] average with standard deviation from replicates) for antisense sRNAs (asRNAs) and 14 TPM for intergenic sRNAs. Second, we used the genome browser IGV to inspect visually and confirm each sRNA. These novel transcripts represented 25 to 30% of the total transcriptome and were 50 to 1,000 nt in length (Fig. 2; see also Table S1). Putative mRNA targets for asRNAs were identified as the *cis* mRNAs encoded on the opposite strands with a minimum overlap of 8 nt, on the basis of IGV confirmation and reports for bacterial asRNAs (34). However, we found in our data that the median overlap between sRNAs and putative *cis*-mRNA targets was 221 nt, with only a small number having a minimum overlap length of 8 nt (see Fig. S2). Analyzing the upstream regions of sRNAs enabled the discovery that 30% of sRNAs contained a BRE and TATA box with centroids at nt −38 and −29 (see Fig. S3). The use of less-conservative parameters (nt −3 and +3) for BRE and TATA box centroids resulted in 70% of sRNAs having transcriptional motifs.

Normalized expression values in RNA-seq analyses are often reported as reads or fragments per kilobase of transcript per million mapped reads (RPKM or FPKM). However, RPKM/FPKM has been shown to be inconsistent for comparisons between samples due to variable transcript lengths. Another expression value, transcripts per million (TPM), was found to be preferable, because it is independent of the mean expressed transcript length and TPM normalization performs better in multiple library comparisons (35–38). To minimize transcript length bias (sRNAs are generally smaller in length than protein-encoding genes) and to compare sRNA and mRNA expression levels, we chose to use TPM in our analysis.

### Noncoding sRNA characterization in H. volcanii under no-challenge conditions.

H. volcanii grown under no-challenge conditions (42°C, complex medium) expressed a total of 1,533 sRNAs after quality control (as described above) (Fig. 2; Table S1), ranging from 49 to 1,000 nt in size and with an average length of 373 nt. A majority of these sRNAs, 1,480 sRNAs (97%), were antisense to coding regions (Fig. 2). The H. volcanii H53 auxotroph genome is 4 Mbp and contains 4,130 genes. The genome constitutes a chromosome stably integrated with small chromosome pHV4 and 2 small chromosomes (pHV1 and pHV3), and it has been cured of plasmid pHV2. A majority of sRNAs (68%) were carried on the chromosome and integrated small chromosome pHV4 (18%). No sRNAs were found on plasmid pHV2, as expected, while sRNAs were carried on the remaining small chromosomes pHV1 (2%) and pHV3 (12%).

We compared mRNA expression to sRNA expression by constructing mRNA sequencing (mRNA-seq) libraries, using the same RNA pool and library preparation as the sRNA-seq libraries (omitting size selection) and calculating transcript expression as TPM (see Fig. S4A). Relative to mRNA expression levels (average, 312.1 ± 1,079.1 TPM), the expression of sRNAs was on average higher (average, 1,107.9 ± 137.6 TPM). We also found that a majority of sRNAs had higher expression levels than mRNAs (sRNA: range, 14.1 to 905,191.0 TPM; median, 108.5 TPM; mRNA: range, 1.0 to 210,162.0 TPM; median, 15.1 TPM). Overall, 75% of sRNAs had expression values less than or equal to 320 TPM, 15% had expression levels similar to those of highly expressed mRNAs, (500 to 10,000 TPM), and 16 sRNAs (sRNAs no. 1771 to 1786) (Table S1) had very robust expression levels with TPMs ranging from 10,000 to 60,000 TPM (Fig. S4A). Lastly, 1 intergenic sRNA (STRG.2577.4), 111 nt in size, exhibited expression levels higher than any mRNA with a TPM of 905,191 (Table S1). Transcript length did not correlate with expression levels, indicating that when we observed sRNAs with low expression levels, it was not an artifact of sequencing (i.e., longer transcripts receiving more read coverage thus skewing coverage based on length) (see Fig. S5). We found that 4 of the 5 most highly expressed sRNAs (TPM > 30,000) were antisense to coding regions (Table S1).

Putative mRNA targets of the most highly expressed asRNAs (≥10,000 TPM) included an IS*4* family transposase (HVO_RS18385), ATP-cob(I)alamin adenosyltransferase (HVO_RS16235), transducer protein Htr36 (HVO_RS15355), pyridoxamine 5′-phosphate oxidase (HVO_RS07060), XerC/D integrase (HVO_RS01885), IS*110* family transposase/pseudoregion (HVO_RS07375), protein translocase TatA (HVO_RS09630), deoxyhypusine synthase (HVO_RS00895), sugar ABC transporter permease (HVO_RS17705), hydrolase (HVO_RS12225), peptidase (HVO_RS08770), RND transporter (HVO_RS14695), and IS*110* family transposase (HVO_RS02445). We do not report putative targets for intergenic sRNAs because of the inherent difficulty in reliably predicting these targets due to unknown degrees of complementarity (i.e., gaps in hybridization between an intergenic sRNA and an mRNA).

### Noncoding sRNAs in H. volcanii under oxidative stress conditions.

H. volcanii under H_2_O_2_-induced oxidative stress conditions expressed 1,227 sRNAs, a 20% decrease in the number of sRNAs compared to that under the no-challenge condition (Fig. 2; Table S1). Despite this decrease, a pattern of sRNA distribution similar to that under the no-challenge condition was observed; more than 94% of sRNAs were antisense and a majority (69%) were carried on the main chromosome. A smaller average length of 337 nt was observed. The overall TPM expression of sRNAs during oxidative stress was similar to that under the no-challenge state, with a decrease in expression level for the single most highly expressed sRNA (STRG.2577.4 in no-challenge, 905,191 TPM; STRG.2983.4 in H_2_O_2_, 810,120 TPM), which was an intergenic sRNA (Fig. S4B; Table S1). Putative targets for the most highly expressed asRNAs (≥10,000 TPM) included both 16S rRNA genes (HVO_RS1301 and HVO_RS18290, two sRNAs), an IS*4* family transposase (HVO_RS18385), an M48 family peptidase (HVO_RS20365), a uracil-DNA glycosylase (HVO_RS09685), Agl cluster protein AglR (HVO_RS20160), a hypothetical protein (HVO_RS20230), transducer protein Htr36 (HVO_RS15355), an ISH3 family transposase/pseudoregion (HVO_RS19935), an IS*4* family transposase (HVO_RS03870), (HVO_RS07060), and an ABC transporter ATP-binding protein (HVO_RS20010). Of these most highly expressed sRNAs, 3 asRNAs (STRG.2050.2, STRG.3974.5, and STRG.4700.1) targeted the same mRNAs, and 3 intergenic sRNAs (STRG.3072.1, STRG.2702.1, and STRG.2983.4) were expressed under both the no-challenge and oxidative stress conditions.

### Regulatory effects and differential expression of sRNAs during oxidative stress.

**FIG 3:**
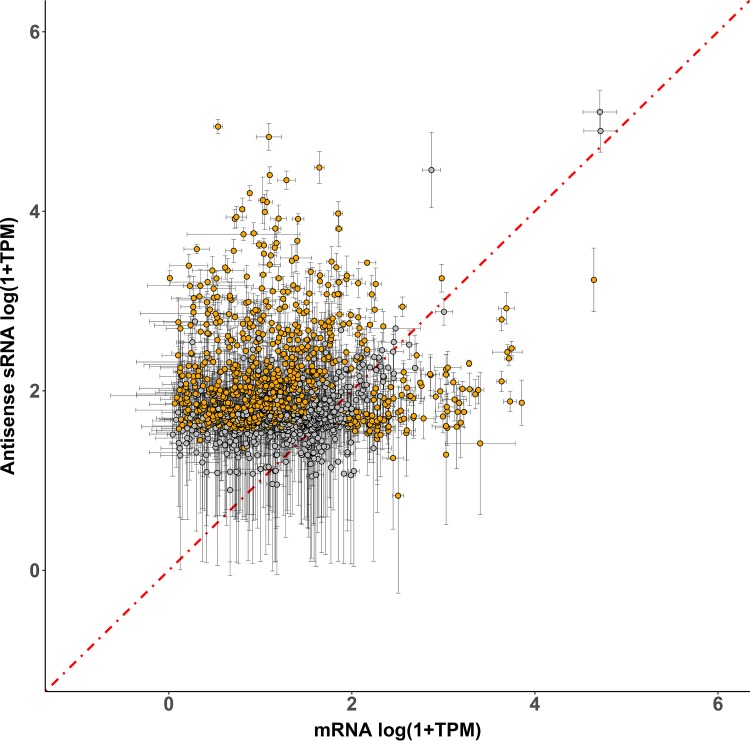
Transcript per million (TPM) expression levels between sRNAs and their putative *cis*-mRNA targets during oxidative stress. Each point represents the TPM ratio between an sRNA and its putative *cis*-mRNA target. TPM values are the averages from sRNA and mRNA replicates with error bars representing standard deviations among replicates. A pairwise *t* test was conducted between sRNA and putative *cis*-mRNA target replicates to infer significant difference in TPM expression between the *cis* pairs. Orange points indicate a *P* value <0.05, and gray points indicate a *P* value >0.05. The red line represents no change in the ratio of sRNA–*cis*-mRNA expression (slope = 1).

To investigate the regulatory effects sRNAs on their target mRNAs, we compared the expression levels (TPM) of all antisense sRNAs against the expression levels (TPM) of the *in silico* predicted putative *cis*-mRNA targets (Fig. 3). We found that a large population of asRNA-mRNA *cis* pairs exhibited lower mRNA target expression than the sRNA, under both experimental conditions (Fig. 3; Table S1). We conducted a pairwise *t* test between these *cis* pairs on expression level differences between sRNAs and putative *cis*-target mRNAs and found that 755 of sRNAs had significantly (*P* < 0.05) higher expression, potentially indicating a negative regulatory relationship between sRNAs and putative *cis*-mRNA targets (Fig. 3).

**FIG 4:**
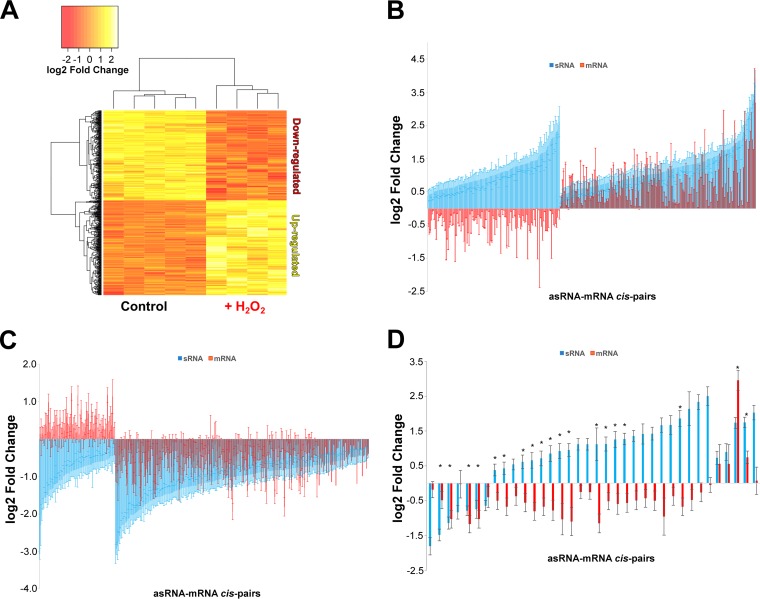
(A) Heat map of log_2_-transformed fold change of differentially expressed antisense sRNAs (asRNAs). (B) Differential expression fold changes of upregulated asRNAs and their putative *cis*-mRNA targets (averages and standard deviation error bars from replicates). (C) Differential expression fold changes of downregulated asRNAs and their putative *cis*-mRNA targets (averages and standard deviation error bars from replicates). (D) Differential expression fold changes of all transposase-targeting asRNAs and their putative *cis*-transposase mRNA targets (averages and standard deviation error bars from replicates). *, both the asRNA and the *cis*-target transposase mRNAs have significant differential expression based on an FDR of <0.05.

To further investigate this negative regulatory relationship between sRNAs and putative mRNA targets, we probed for differentially expressed sRNAs between the no-challenge and the oxidative stress conditions. Candidate sRNAs were considered significantly up- or downregulated by oxidative stress using a false-discovery rate (FDR) of less than 5%. Using this statistical framework, we identified a core set of differentially expressed sRNAs specific to oxidative stress (Fig. 4A). Both intergenic and antisense sRNAs were differentially expressed. Of the intergenic sRNAs, 48 were significantly differentially expressed (FDR < 0.05), with 23 upregulated and 25 downregulated (see Fig. S6; Table S2). Of these upregulated intergenic sRNAs, 79% had an increase in expression greater than or equal to a fold change of 2 (log_2_ fold change = 1) during oxidative stress, with the most upregulated intergenic sRNA (STRG.277.2) having a 16-fold increase. On the other hand, a majority of downregulated intergenic sRNAs had large fold changes in expression (≤2-fold change), and 5 exhibited very robust downregulation (≤−4-fold change). A total of 605 antisense sRNAs were significantly (FDR < 0.05) differentially expressed. These sRNAs were either upregulated (309) or downregulated (296) during oxidative stress, indicating two populations of antisense sRNAs (Fig. 4A; Table S2). Fifty percent (302 sRNAs) of these differentially expressed sRNAs demonstrated a fold change in expression of ±2 or greater; the most upregulated sRNA had a fold change of 15 and the most downregulated sRNA had a fold change of −9, indicating a role for these sRNAs in the cellular response to oxidative stress. More antisense sRNAs were upregulated with a fold change in expression of 4 or greater (28 sRNAs) than were downregulated (20 sRNAs). We then compared differential expression levels between all significantly upregulated antisense sRNAs and their putative *cis*-mRNA targets and found that a population (133 sRNAs [22%]) of these upregulated antisense sRNAs had putative mRNA targets that were downregulated during oxidative stress (Fig. 4B). For example, during oxidative stress, 36 upregulated antisense sRNAs targeted transposase mRNAs, and 24 of these antisense sRNAs had putative *cis*-target transposase mRNAs downregulated (Fig. 4D). While a smaller subset of significantly (FDR < 0.05) downregulated antisense sRNAs had their putative *cis*-mRNA target upregulated during oxidative stress (72 [11%]), it also indicated a correlative potential negative regulatory effect (Fig. 4C).

**FIG 5:**
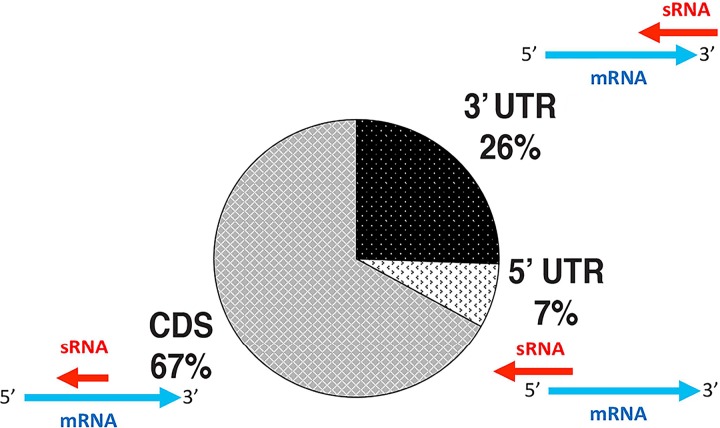
Distribution of binding regions for antisense sRNAs. UTR, untranslated region; CDS, coding sequence.

**FIG 6:**
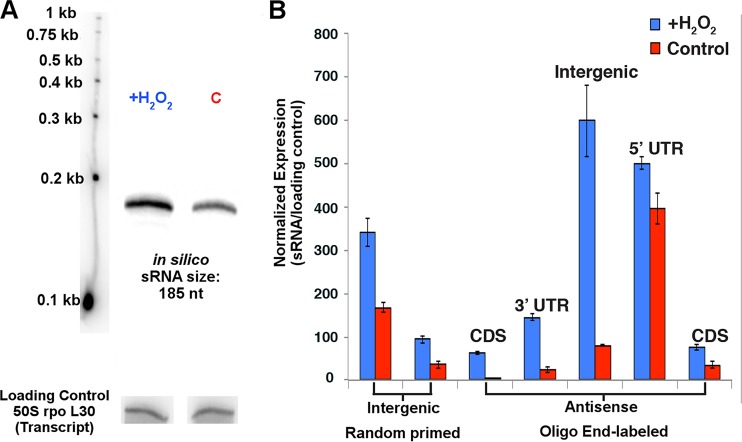
Validation of differentially expressed sRNAs by Northern blots. (A) Representative Northern blot confirming sizes and differential expression patterns of an intergenic sRNA during oxidative stress. (B) Quantification of Northern blots confirming the expression of intergenic sRNAs (random primed labeling) and strand specificity of sRNAs (oligonucleotide labeling). All classes of sRNAs were confirmed: antisense (5′ UTR, 3′ UTR, CDS) and intergenic sRNAs.

Oxidative stress-responsive asRNAs were predicted to overlap both the 5′ and 3′ UTRs of mRNAs (Fig. 5). We found that 7% of asRNAs overlapped at the 5′ UTRs and 26% overlapped at the 3′ UTRs. However, the majority of the asRNAs (67%) overlapped the coding sequences (CDSs) of mRNAs rather than the UTRs, which has not been previously reported (Fig. 5). We calculated that, on average, the overlap between sRNAs and their putative *cis*-target mRNAs was 265 nt, the range was 8 to 992 nt, and the peak overlap was between 150 and 200 nt (Fig. S2). Using Northern blot analysis, we recapitulated the *in vivo* differential expression patterns of selected candidate sRNAs, further confirming the transcript sizes and differential expression levels for oxidative stress sRNAs (STRG.3823.1, -4700.1, -8.6, -277.2, -3733.1, -2983.4, and -4213.4) (Fig. 6A and B). We also showed that the strandedness (the strand on which the sRNA was carried) predicted by our sRNA-seq analysis was confirmed by our *in vivo* data using oligonucleotide probe Northern blotting of 5′ UTRs, 3′ UTRs, CDS antisense sRNAs, and intergenic sRNAs (Fig. 6B).

### Target enrichment of sRNAs.

**FIG 7:**
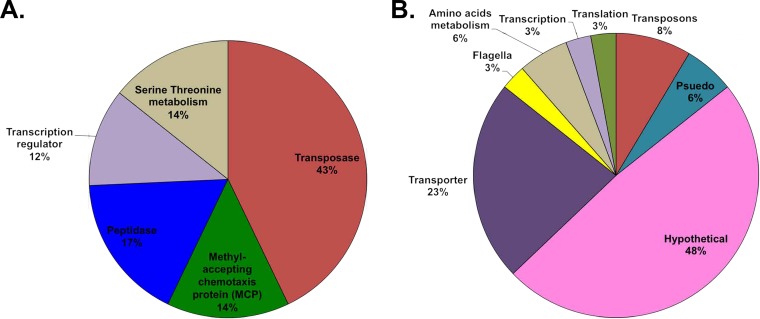
Gene ontology enrichment analysis identifying the functional classification of gene targets of sRNAs during oxidative stress. (A) Enriched target gene functions for upregulated sRNAs. (B) Enriched target gene functions for downregulated sRNAs.

We identified *in silico* targets for the differentially expressed oxidative-stress responsive antisense sRNAs. Genes for the putative target mRNAs were categorized according to cellular function by using archaeal Cluster of Orthologous Genes (arCOGs) and according to pathways by using gene ontologies (GO) from Database for Annotation, Visualization, and Integrated Discovery (DAVID). For sRNAs upregulated during H_2_O_2_ stress, we found a functional enrichment of target genes encoding transposases, involved in chemotaxis methyl-receptor signaling and in transcriptional regulation (transcription factors) (*P* < 0.05). Genes from many other pathways that were not enriched were also the targets of antisense sRNAs, including peptidase activity genes and serine and threonine biosynthesis genes (Fig. 7A). Twenty-three of these sRNAs targeted transposase genes. Each of these transposase mRNAs was downregulated, while their cognate sRNA was upregulated, and the sRNA was always located at the 5′ UTR of its target. Most transposases belonged to the IS family of transposases, except for one DDE transposase. Three transcription factor families (IclR, ArcR, and AsnC) were also targeted by asRNAs. A functional enrichment gene ontology analysis found that downregulated sRNAs targeted genes involved in membrane transport (ABC transporters) and the biosynthesis of secondary metabolites, as well as hydrolases (Fig. 7B). A significant proportion of enriched targets for both up- and downregulated sRNAs were genes encoding hypothetical proteins.

### mRNA transcriptional response to oxidative stress in H. volcanii.

**FIG 8:**
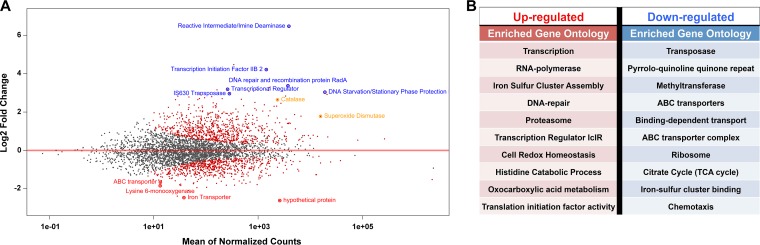
Distribution of differentially expressed genes during oxidative stress in H. volcanii. (A) MA plot of differentially expressed genes; each point represent a gene. Significant (FDR < 5%) differentially expressed sRNAs are color coded as upregulated (blue) or downregulated (red) and known oxidative stress response genes (yellow). (B) Gene function for the most up- and downregulated sRNAs.

To determine the transcriptional landscape of mRNAs during oxidative stress, especially for mRNAs that were predicted targets of sRNAs, we sequenced rRNA-depleted mRNA-seq libraries in parallel with the previously described sRNA-seq libraries (derived from the same pool of total RNA). During H_2_O_2_-induced oxidative stress, one-fourth of all genes (1,176) were significantly differentially expressed with a false-discovery rate of less than 5% (see Table S4). Both catalase and superoxide dismutase, known ROS detoxification enzymes, were upregulated at the mRNA level, thus validating our experimental approach and characterizing H. volcanii response to oxidative stress at the transcriptional level (Fig. 8). A GO enrichment analysis (DAVID) was used to identify what pathways were enriched with differentially expressed genes during oxidative stress. The most enriched (*P* < 0.05) upregulated genes were involved in transcription, including various transcription factor families, all of the RNA polymerase subunit genes, and transcription initiation factors. Other enriched (*P* < 0.05) upregulated pathways were involved in iron-sulfur cluster assembly, DNA topological change (topoisomerase), proteasomes, cell redox homeostasis, histidine metabolism, and 2-oxocarboxylic acid metabolism. The most upregulated gene was a reactive intermediate/imine deaminase with a fold change expression increase of 84. The most enriched (*P* < 0.05) downregulated genes were Tn*5*-like IS*4* transposases. Other enriched (*P* < 0.05) downregulated pathways were pyrroloquinoline quinone (PQQ) proteins, tetrapyrrole methyltransferases, and ABC transporters. Only two genes had a downregulation of <−4-fold, and these were an iron transporter and lysine 6-monooxygenase.

## DISCUSSION

Previous studies of sRNAs in Archaea revealed the abundance of sRNAs within the third domain of life and have been pivotal in establishing a working hypothesis on archaeal sRNA functionality. These studies have been limited to (i) microarray studies that do not enable *de novo* discovery of sRNAs, (ii) differential RNA-seq (dRNA-seq) approaches, which select only for primary transcripts and do not provide length (nt) information nor expression information (only coverage), and (iii) individual sRNA studies, which do not give a holistic view of the pathways being regulated within the cell. Using a custom strand-specific sRNA-seq library preparation and analysis pipeline, we have developed a method to perform high-throughput analysis of the sRNA transcriptional landscape, expression, and regulatory effects, and to identify regulated gene pathways in response to environmental stressors within the Archaea. In this study, we propose that sRNA-mediated transcriptional regulation is key in regulating stress responses to environmental challenges, such as oxidative stress, in the haloarchaea. sRNAs have the potential to carry out large-scale regulation of genes involved in the oxidative stress response, resulting in increased resistance to extreme environmental stressors.

The discovery that sRNAs comprised nearly one-third of the total transcriptome of H. volcanii and included basal transcriptional promoters, under both no-challenge and oxidative stress conditions, suggests that sRNAs have an important functional role under a variety of environmental conditions. We discovered thousands of sRNAs expressed in H. volcanii, with the majority being antisense to genes, indicating that antisense transcription was ubiquitous within the cell. This is in stark contrast to most of the literature reporting that a majority of sRNAs discovered in Archaea were intergenic (9, 13, 22, 39). This discrepancy is likely due to previous studies being limited to microarray approaches. Indeed, a recent study using directional RNA-seq (dRNA-seq) to map all transcription start sites (TSS) in H. volcanii found thousands of novel TSS, with 1,244 of these TSS being antisense to mRNAs (24). Most of the TSS (75%) of the sRNAs we discovered in H. volcanii had the same TSS (±10 nt) as those found in the dRNA-seq study by Babski et al. (24), further confirming our results. This underlines the importance of HTS studies, especially strand-specific RNA-seq such as in our study, to discover the full extent of antisense sRNA expression in Archaea.

Our finding suggests that a priori *cis*-acting sRNAs may play a larger role than *trans*-acting sRNAs within the cell. It should not be overlooked that the difficulty in finding *in silico* targets for intergenic sRNAs, because these sRNAs do not form 100% complementarity with their targets, might suggest that they have multiple mRNA targets. Antisense and intergenic sRNAs are broad classifications used in the archaeal small noncoding RNA field, but our data revealed that further classifications can be made on the basis of sRNA-mRNA binding characteristics (5′ UTR, 3′ UTR, and CDS), differential expression, and regulatory effects. We found that only a small fraction of asRNAs targeted the 5′ UTRs of mRNAs, which is in concurrence with work demonstrating that most mRNAs in H. volcanii are leaderless (lacking a 5′ UTR). A majority of the 5′ UTR-binding sRNAs targeted transposons, providing further evidence that they may constitute their own class of sRNAs. Within this context, 3′ UTR-binding sRNAs should also be considered another class of sRNAs, resembling eukaryotic sRNAs, which likely lead to the degradation of their target transcripts. The majority of the asRNAs we identified in H. volcanii had 100% complementarity within the CDS of their target mRNAs. This is the first report of such a finding in any domain of life and might constitute an attribute unique to archaeal sRNAs. We could not identify any “seed” binding region for these CDS-binding sRNAs, indicating that they likely have full occupancy on the mRNA. It is worth noting that there were only a few instances (<20) where sRNAs overlapped more than the full length of the target mRNA (i.e., the asRNA is longer than the mRNA) or overlapped multiple *cis*-mRNA targets (i.e., the asRNA overlaps the 3′ UTR of one mRNA and the 5′ UTR of an adjacent mRNA), which is novel in the Archaea.

Most H. volcanii sRNAs had a normalized expression value of 200 TPM or less, indicating that sRNA transcripts are relatively abundant in the cell. The use of a stringent thresholding approach resulted in a smaller number of more highly expressed sRNAs but avoided potentially reporting false positives with low TPM values or transcriptional noise. In comparison, most mRNAs within H. volcanii had TPM values of 20 or less. We found a population of antisense sRNAs with significantly higher expression than their putative *cis*-mRNA targets, suggesting a potential negative regulatory role in sRNA-mRNA interactions (Fig. 3 and 4B) (40–42). This trend extended to many asRNAs (under both no-challenge and oxidative stress conditions), down to sRNAs with expression levels of 40 TPM. Stronger evidence for a negative regulatory effect lies with a population of upregulated sRNAs. A group of significantly upregulated asRNAs had target mRNAs that were downregulated during oxidative stress, indicating these asRNAs could potentially negatively regulate mRNA targets at the transcript level. Whether this negative regulation is occurring during transcription initiation/elongation or if these asRNAs are causing mRNA degradation is currently unknown. All the asRNAs targeting transposons at the 5′ UTR were upregulated, and the transposon mRNAs were downregulated (Fig. 4D), suggesting that these asRNAs might have a similar mechanistic function (6, 43, 44). If indeed these asRNAs are negatively regulating their target mRNAs in H. volcanii, we expected to find that downregulated asRNAs have upregulated target mRNAs. Some downregulated asRNA targets exhibited this pattern, further supporting a potential negative regulation (Fig. 4C). Many mRNA targets of both up- and downregulated asRNAs exhibited the same regulatory pattern, suggesting a potential nonnegative regulatory role for a group of asRNAs (Fig. 4). Alternative hypotheses, reflecting the complexity of transcriptional regulation in the Archaea, can be formed: (i) these asRNAs may have a positive regulatory effect, such as stabilizing target mRNAs and masking them from degradation as seen with an intergenic sRNA (itsRNA) in M. mazei (23), (ii) *trans*-acting intergenic sRNAs might be targeting these mRNAs, negatively regulating them, and (iii) some may have an unknown function (23).

The most enriched negatively regulated sRNA targets were transposases, chemotaxis proteins, and transcription factors. It has been shown that transposons are opportunistic, and under stress conditions, they can wreak havoc by hopping around in the genome causing double-strand breaks and hence need to be silenced (6, 43, 44). A functional enrichment of IS*4* transposon genes that are downregulated during oxidative stress supports our observation that the upregulated sRNAs can potentially negatively regulate transposons and suggests that transposon activity is tightly regulated during oxidative stress in H. volcanii. sRNA-mediated regulation of chemotaxis transducer proteins during oxidative stress suggests interesting implications in sensing ROS and motility. H. volcanii expresses genes coding for a flagellum analog named “archaellum,” which are organized into an operon and are regulated by a network of regulators called the archaellum regulatory network (arn) (identified in crenarchaea) (45, 46). The regulation of these motility genes is still under investigation and so far is restricted to a few examples, such as under H_2_ or nitrogen limitation conditions in Methanococcus jannaschii and Methanococcus maripaludis (46–49). No direct transcriptional regulators of the archaellum have been identified in Euryarchaeota, but the deletion of archaellin genes, the presence of the H-domain set of type IV pillins, and Agl proteins have been shown to affect the assembly of the archaellum in H. volcanii (46, 50–52). Integral to how microorganisms maintain homeostasis in stressful and fluctuating environments are the gene regulatory networks composed of interacting regulatory transcription factors and their target gene promoters (53). Our discovery that sRNAs are targeting transcription factors provides evidence that sRNAs are likely deeply interlaced within complex gene regulatory networks of H. volcanii, and these sRNAs are key to maintaining homeostasis during environmental stresses such as oxidative stress. Many mRNA targets of differentially regulated sRNAs were hypothetical proteins, indicating that much remains to be elucidated in this organism.

Our whole transcriptional analysis demonstrated that close to one-third of the genes (∼1,100 [30%]) in H. volcanii were differentially regulated during constant H_2_O_2_-induced oxidative stress at ∼80% survival, which is in agreement with the transcriptional response of H. salinarum during constant H_2_O_2_-induced (929 genes [38%]) and paraquat-induced (1,099 genes [45%]) oxidative stresses at ∼80% survival (2). This indicates that transcriptional regulation is crucial in order to mount this oxidative stress response via gene activation and repression. Two single-stranded DNA binding proteins (RpaB and RpaC) were found necessary for the increased survival of H. volcanii under ionizing radiation (a proxy for desiccation) and UV radiation, stressors that both cause oxidative stress (54, 55) (J. DiRuggiero, unpublished data). In H. salinarum, Rpa operons were upregulated under ionizing radiation as well and contributed to resistance (56, 57). In conjunction with previous findings, we observed that two of the most upregulated genes during H_2_O_2_ oxidative stress were RpaB1 (HVO_RS10725, 23-fold change) and RpaB2 (HVO_RS06105, 8-fold change), confirming their roles in oxidative stress resistance in H. volcanii and likely other haloarchaea. One gene, a reactive intermediate/imine deaminase RidA-homolog (HVO_RS12485), was upregulated orders of magnitude more than any other gene. The encoded protein is known to be involved in the synthesis of branched-chain amino acids by speeding up the IlvA-catalyzed deamination of threonine into 2-ketobutyrate (58, 59). Previous work has shown that in the presence of reactive chlorine species (RCS), such as HOCl, imine deaminase seemed to inhibit IlvA activity suggesting that imine deaminase may have a different function in the presence of RCS (58, 60). Further studies found that imine deaminase can sense RCS and, in doing so, becomes a chaperone that prevents protein aggregation (58). Reactive oxygen species in hypersaline environments produce RCS (61). In addition, ROS cause extensive irreversible protein damage such as carbonylation, which in turn causes protein aggregation (62, 63). This reactive intermediate/imine deaminase is the most upregulated protein-encoding gene, suggesting that it may be playing a similar chaperone role to prevent protein aggregation, by sensing either ROS or RCS produced by H_2_O_2_ (58, 60).

This study is the first to report on the transcriptional response of H. volcanii to oxidative stress, and while we found similar responses to H_2_O_2_ exposure as previously reported for H. salinarum (2), further validating our work and providing evidence that haloarchaea have evolved similar strategies to survive their environmental stresses, we also found responses that were unique to H. volcanii. Similarities to H. salinarum include the upregulation of genes encoding ROS scavenging proteins (catalase and superoxide dismutase) and iron sulfur assembly proteins (SufB and SufD), as well as proteasome genes, indicating high protein turnover, and many DNA repair genes (2). Most of the downregulated genes were involved with metabolism, such as sugar/phosphate/peptide ABC transporters, electron carriers (halocyanin), and tricarboxylic acid (TCA) cycle enzymes, possibly to halt growth until damage is repaired (2, 64). The most downregulated gene was for an iron ABC transporter, most likely to limit further production of ROS via Fenton reactions (2). Of the unique responses to oxidative stress in H. volcanii, we found that genes encoding all of the RNA polymerase subunits and transcription elongation factors and seven basal transcription initiation factors (HVO_RS12755, 18.4-fold change; HVO_RS01380, 8.6-fold; HVO_RS09745, 6.5-fold; HVO_RS01840, 2.1-fold; HVO_RS05475, 1.74-fold; HVO_RS11835, 1.5-fold; HVO_RS05475, 1.3-fold change) were significantly upregulated in response to oxidative stress. The increase in sRNAs during oxidative stress could be attributed to this increase in transcription machinery. The majority of the 30S and 50S ribosomal subunits were downregulated, in contrast to that in H. salinarum. The upregulations of genes coding for histidine biosynthesis and catabolism into glutamate and 2-oxocarboxylic acid metabolism were unknown to be involved in the oxidative stress response, which further demonstrates there are still more mechanisms to uncover for oxidative stress resistance. RosR was identified as a global transcriptional regulator in H. salinarum, and it was strongly upregulated during oxidative stress (5). RosR demonstrated no differential expression during oxidative stress in H. volcanii, indicating that it may play another role in this organism. Cell cycle genes (*parA* and *cdc6*) involved in chromosome segregation (65) were downregulated, further suggesting that division is being arrested (halting growth) in order to repair damage.

In this study, we showed for the first time that small noncoding RNAs are specifically associated with the oxidative stress response in archaea. During oxidative stress, asRNAs were prevalently transcribed, comprising nearly 30% of the transcriptome of H. volcanii, and many upregulated asRNAs imparted a correlative negative regulatory effect on putative target mRNAs. These results support the hypothesis that some asRNAs in Archaea behave similarly to *cis*-acting bacterial sRNAs and eukaryotic siRNAs, which negatively regulate mRNAs by sharing extensive complementarity and facilitating RNA degradation (66, 67). The precise mechanism(s) of sRNA-mRNA mediated regulation, in particular, whether proteins are required to complex with sRNAs in order to mediate gene regulation, such as in Bacteria (Hfq) and Eukarya (Ago), remains to be elucidated. We also identified several classes of asRNAs, on the basis of their mRNA-binding patterns (3′ UTR, 5′ UTR, and CDS) and showed that CDS targeting of mRNAs was the predominant mode of action for sRNA hybridization. The mechanistic differences between these classes of sRNAs, as well as the regulatory roles of sRNAs in archaea and their functional importance in adaption to extreme environments, still need to be investigated.

## MATERIALS AND METHODS

### Culture growth conditions.

H. volcanii auxotrophic strain H53 (Δ*pyre2* Δ*trpA*) was used for all experiments. Culturing in liquid and solid media was done in rich medium (Hv-YPC) at 42°C and with shaking at 220 rpm (Amerex Gyromax 737) (68). Uracil and tryptophan were added to a final concentration of 50 μg/ml each.

### Oxidative stress exposure.

We exposed 5 biological replicates of H. volcanii strain H53 liquid cultures to the oxidative stress agent H_2_O_2_. Initially, cultures were grown in 80 ml of Hv-YPC under optimal conditions to an optical density (OD) of 0.4 (mid-exponential phase). To ensure homogeneity, each replicate was subsequently split into two 40-ml cultures, one used for the no-challenge (control) condition and the other for the oxidative stress condition. For the latter condition, 2 mM H_2_O_2_ (80% survival rate, previously determined) was directly added to the cultures followed by a 1-h incubation at 42°C with shaking at 220 rpm. Cultures were then rapidly cooled down and centrifuged at 5,000 × *g* for 5 min, and the pellets were resuspended in 18% seawater. The cell suspensions were then transferred to 1-ml tubes and centrifuged at 6,000 × *g* for 3 min, and the pellets were flash frozen and stored at −80°C until ready for RNA extraction. Control no-challenge culture replicates were processed in the same manner without the addition of H_2_O_2_.

### Oxidative stress survival curves.

The assessment of survival in H. volcanii under oxidative stress conditions was conducted using microdilution plating as described previously (7). The counts from replicates were averaged and the standard deviations calculated. Survival was calculated as the number of viable cells following H_2_O_2_ treatment divided by the number of viable untreated cells and graphed with standard error bars.

### RNA extraction.

Total RNA was extracted using the Zymo Quick-RNA Miniprep kit with the following modifications: after the addition of RNA lysis buffer to the frozen cell pellets, the cells were processed with 23-gauge needles and syringes to ensure complete cell lysis. H. volcanii liquid cultures are slimy and viscous; thus, to increase cellular lysis, 23-gauge needles and syringes were used to break down the cell pellets. Total RNA was then extracted according to the standard kit protocol.

### Small RNA-sequencing library preparation.

Total RNA for each biological replicate and condition was size selected by denaturing polyacrylamide gel electrophoresis. Twenty micrograms of total RNA was loaded onto a 7% denaturing urea polyacrylamide gel (SequaGel; National Diagnostics) in 0.5× Tris-borate-EDTA (TBE) buffer and run at a constant power of 30 W until the bromophenol blue bands reached the bottom of the gel. The gel was stained with SYBR Gold and visualized on a blue light box, and bands in the 50 to 500 nucleotide range, as indicated by the RNA Century Marker plus ladder (Thermo Fisher), were excised. Small RNAs (sRNA) were eluted by rotating overnight in 1.2 ml 0.3 M NaCl and were ethanol precipitated and DNase I (NEB) treated (37°C for 2 h) as previously described (69). Strand-specific libraries were prepared using the SMART-seq Ultralow RNA input kit (TaKaRa), and insert sizes were checked with the Bioanalyzer RNA pico kit (Agilent). Paired-end sequencing (2 × 150 bp) was carried out on the Illumina HiSeq 2500 platform at the Johns Hopkins University Genetic Resources Core Facility (GRCF).

### mRNA-sequencing library preparation.

Individual mRNA-seq libraries were made from the same RNA pools as the sRNA-seq libraries above. Total RNA was rRNA depleted using the Ribo-zero bacteria kit (Illumina) and sequenced using the sRNA-seq library preparation method described above but omitting the size selection by denaturing urea polyacrylamide gel electrophoresis.

### sRNA- and RNA-seq read quality control and reference-based read mapping.

The assessment of the quality of each sequencing library read was conducted using fastqc. The program trim galore was used with base settings to trim adapter sequences from reads and to filter out low phred score reads (<20). Short-length reads were preserved. Reads from each replicate were aggregated together per condition to get a set of consensus sRNAs and were mapped against the H. volcanii NCBI RefSeq genome (taxonomy identification [taxid] 2246; 1 chromosome, 4 plasmids) using the hisat2 aligner with strand-specific options turned on and splice aware options turned off, in paired-end mode (70).

### sRNA- and RNA-seq transcriptome assembly.

The reference-based alignments were assembled into transcriptomes using the program stringtie to build full-length transcripts and calculate coverage and expression values (TPM). The assembly was guided by a gene annotation file from the H. volcanii DS2 (NCBI RefSeq taxid 2246) genome to build transcripts and annotate them as either a gene or novel transcript (71). The minimum distance between reads for transcript assembly was specified at 30 nucleotides. gffcompare under default options was used to compare the assembled transcriptomes against the gene annotation file from H. volcanii DS2 (NCBI RefSeq taxid 2246) to annotate transcripts as genes or noncoding RNA (antisense or intergenic) (72, 73). In-house python scripts were used to bin transcripts that were annotated as genes, transcripts annotated as antisense (classified as a noncoding region opposite from a coding region), and transcripts annotated as intergenic (classified as a noncoding region between two coding regions), and antisense sRNAs were subsequently binned as 3′ UTR, 5′ UTR, or CDS overlapping.

### sRNA- and RNA-seq differential expression analysis.

We used a read count-based differential expression analysis to identify differentially expressed sRNAs during oxidative stress. The program featureCounts was used to rapidly count reads that map to the assembled sRNA transcripts (described above) (74). featureCounts was run with strand-specific options on, paired-end mode on, and multimapping off (74). The read counts were then used in the R differential expression software package DESeq2 (75). Briefly, read counts were converted into a data matrix and normalized by sequencing depth and geometric mean. Differential expression was calculated by finding the difference in read counts between the no-challenge state (control) normalized read counts and the oxidative stress normalized read counts (75). The differentially expressed sRNAs were filtered on the basis of the statistical parameter of false-discovery rate (FDR), and those that were equal to or under an FDR of 5% were classified as true differentially expressed sRNAs.

### *In silico* validation of sRNAs.

Differentially expressed sRNAs were validated by two *in silico* methods: (i) visualization of transcripts and (ii) open reading frame protein homology search. In the first method, transcriptomes for each replicate and condition were visualized on the Integrated Genome Viewer (IGV) against the H. volcanii (NCBI RefSeq taxid 2246) genome and annotation (76). The sRNA transcript coordinates were used to locate putative sRNAs, and if it was found within an operon, it was eliminated from further analysis. In the second method, blastx (default parameters) was used to search for protein and domain homology for each sRNA, and those that had significant homology with known proteins or domains were eliminated from further analysis (77).

### Regulatory element motif identification of sRNAs.

One hundred nucleotides upstream and downstream from the sRNA transcript start and stop coordinates were extracted using in-house python scripts. These regions were searched for transcription motifs (BRE and TATA box) using multiple sequence alignments, visualization with WebLogo (default parameters), and motif searching with MEME and CentriMo (default parameters) (78, 79).

### *In vivo* validation of sRNAs by Northern blot analysis.

Twenty micrograms of total RNA and [^32^P]ATP end-labeled Century-Plus RNA markers was loaded onto 5% denaturing urea polyacrylamide gels (SequaGel; National Diagnostics) and run at 30 W for 1.5 h to ensure well-spaced gel migration from 50 to 1,000 nucleotides (nt). Gels were transferred onto Ultrahyb nylon membranes and hybridized with 2 types of probes. For lowly expressed sRNAs, we probed with [γ-^32^P]dATP randomly primed amplicons generated with custom primers based on sRNA transcript genomic coordinates as determined by the sRNA-seq *in silico* analysis. Probe primers were at a minimum of 10 nt inward from the predicted genomic coordinates (start and stop) to ensure accurate transcript detection. Hybridizations were performed at 65°C. To determine strandedness of sRNAs, we used [α-^32^P]dATP end-labeled oligonucleotide probes (20 to 23 nt) that were antisense to sRNAs. Hybridizations were at 42°C. The rpl30 protein (HVO_RS16975) transcript was used as a loading control for differential expression calculation, because it was not differentially expressed under oxidative stress in this RNA-seq data set. Differential expression was calculated using ImageJ.

### Gene ontology enrichment analysis of mRNA-targets.

NCBI gene names for all mRNA targets of antisense sRNAs were uploaded into Database for Annotation, Visualization, and Integrated Discovery (DAVID) to determine the pathways and gene ontologies targeted by sRNAs.

### Accession number(s).

All raw reads and processed data from these experiments are available at NCBI under BioProject PRJNA407425. Illumina raw sequence data (.fastq) for each replicate and condition are deposited in NCBI Sequence Read Archive with accession number SRP117726.

## Supplementary Material

Supplemental material

## References

[1] Robinson CK, Wierzchos J, Black C, Crits-Christoph A, Ma B, Ravel J, Ascaso C, Artieda O, Valea S, Roldan M, Gomez-Silva B, DiRuggiero J 2015 Microbial diversity and the presence of algae in halite endolithic communities are correlated to atmospheric moisture in the hyper-arid zone of the Atacama Desert. Environ Microbiol 17:299–315. doi:10.1111/1462-2920.12364. 24372972

[2] Kaur A, Van PT, Busch CR, Robinson CK, Pan M, Pang WL, Reiss D, DiRuggiero J, Baliga NS 2010 Coordination of frontline defense mechanisms under severe oxidative stress. Mol Syst Biol 6:393. doi:10.1038/msb.2010.50. 20664639 PMC2925529

[3] Imlay JA 2008 Cellular defenses against superoxide and hydrogen peroxide. Annu Rev Biochem 77:755–776. doi:10.1146/annurev.biochem.77.061606.161055. 18173371 PMC3057177

[4] Crits-Christoph A, Gelsinger DR, Ma B, Wierzchos J, Ravel J, Ascaso C, Artieda O, Davila A, DiRuggiero J 2016 Functional analysis of the archaea, bacteria, and viruses from a halite endolithic microbial community. Environ Microbiol 18:2064–2077. doi:10.1111/1462-2920.13259. 26914534

[5] Sharma K, Gillum N, Boyd JL, Schmid A 2012 The RosR transcription factor is required for gene expression dynamics in response to extreme oxidative stress in a hypersaline-adapted archaeon. BMC Genomics 13:351. doi:10.1186/1471-2164-13-351. 22846541 PMC3443676

[6] Whitehead K, Kish A, Pan M, Kaur A, Reiss DJ, King N, Hohmann L, DiRuggiero J, Baliga NS 2006 An integrated systems approach for understanding cellular responses to gamma radiation. Mol Syst Biol 2:47. doi:10.1038/msb4100091. 16969339 PMC1681521

[7] Robinson CK, Webb K, Kaur A, Jaruga P, Dizdaroglu M, Baliga NS, Place A, DiRuggiero J 2011 A major role for nonenzymatic antioxidant processes in the radioresistance of *Halobacterium salinarum*. J Bacteriol 193:1653–1662. doi:10.1128/JB.01310-10. 21278285 PMC3067647

[8] Webb KM, Wu J, Robinson CK, Tomiya N, Lee Y, DiRuggiero J 2013 Effects of intracellular Mn on the radiation resistance of the halophilic archaeon *Halobacterium salinarum*. Extremophiles 17:485–497. doi:10.1007/s00792-013-0533-9. 23532412

[9] Sharma A, Grichenko O, Matrosova VY, Hoeke V, Klimenkova P, Conze IH, Volpe RP, Tkavcb R, Gostinþar G, Gunde-Cimerman N, DiRuggiero J, Shuryakg I, Ozarowskih A, Hoffmana BM, Daly MJ 2017 Across the tree of life, radiation resistance is governed by antioxidant Mn2+, gauged by paramagnetic resonance. Proc Nat Acad Sci U S A 114:E9253–E9260. doi:10.1073/pnas.1713608114. PMC5676931 29042516

[10] Morris KV, Mattick JS 2014 The rise of regulatory RNA. Nat Rev Genet 15:423–437. doi:10.1038/nrg3722. 24776770 PMC4314111

[11] Altuvia S, Weinstein-Fischer D, Zhang A, Postow L, Storz G 1997 A small, stable RNA induced by oxidative stress: role as a pleiotropic regulator and antimutator. Cell 90:43–53. doi:10.1016/S0092-8674(00)80312-8. 9230301

[12] Cech TR, Steitz JA 2014 The noncoding RNA revolution-trashing old rules to forge new ones. Cell 157:77–94. doi:10.1016/j.cell.2014.03.008. 24679528

[13] Marchfelder A, Fischer S, Brendel J, Stoll B, Maier LK, Jager D, Prasse D, Plagens A, Schmitz RA, Randau L 2012 Small RNAs for defence and regulation in archaea. Extremophiles 16:685–696. doi:10.1007/s00792-012-0469-5. 22763819 PMC3432209

[14] Prasse D, Ehlers C, Backofen R, Schmitz RA 2013 Regulatory RNAs in archaea: first target identification in methanoarchaea. Biochem Soc Trans 41:344–349. doi:10.1042/BST20120280. 23356309

[15] Altuvia S 2004 Regulatory small RNAs: the key to coordinating global regulatory circuits. J Bacteriol 186:6679–6680. doi:10.1128/JB.186.20.6679-6680.2004. 15466017 PMC522215

[16] Gottesman S, Storz G 2011 Bacterial small RNA regulators: versatile roles and rapidly evolving variations. Cold Spring Harb Perspect Biol 3:a003798. doi:10.1101/cshperspect.a003798. 20980440 PMC3225950

[17] Fischer S, Benz J, Spath B, Maier LK, Straub J, Granzow M, Raabe M, Urlaub H, Hoffmann J, Brutschy B, Allers T, Soppa J, Marchfelder A 2010 The archaeal Lsm protein binds to small RNAs. J Biol Chem 285:34429–34438. doi:10.1074/jbc.M110.118950. 20826804 PMC2966057

[18] Fischer S, Benz J, Spath B, Jellen-Ritter A, Heyer R, Dorr M, Maier LK, Menzel-Hobeck C, Lehr M, Jantzer K, Babski J, Soppa J, Marchfelder A 2011 Regulatory RNAs in *Haloferax volcanii*. Biochem Soc Trans 39:159–162. doi:10.1042/BST0390159. 21265765

[19] Heyer R, Dorr M, Jellen-Ritter A, Spath B, Babski J, Jaschinski K, Soppa J, Marchfelder A 2012 High throughput sequencing reveals a plethora of small RNAs including tRNA derived fragments in *Haloferax volcanii*. RNA Biol 9:1011–1018. doi:10.4161/rna.20826. 22767255 PMC3495736

[20] Schmitz-Streit R, Jäger D, Jellen-Ritter A, Babski J, Soppa J, Marchfelder A 2011 Archaea employ small RNAs as regulators, p 131–145. *In* Hess WR, Marchfelder A (ed), Regulatory RNAs in prokaryotes. Springer Verlag, Vienna, Austria.

[21] Soppa J, Straub J, Brenneis M, Jellen-Ritter A, Heyer R, Fischer S, Granzow M, Voss B, Hess WR, Tjaden B, Marchfelder A 2009 Small RNAs of the halophilic archaeon *Haloferax volcanii*. Biochem Soc Trans 37:133–136. doi:10.1042/BST0370133. 19143617

[22] Straub J, Brenneis M, Jellen-Ritter A, Heyer R, Soppa J, Marchfelder A 2009 Small RNAs in haloarchaea: identification, differential expression and biological function. RNA Biol 6:281–292. doi:10.4161/rna.6.3.8357. 19333006

[23] Prasse D, Förstner KU, Jäger D, Backofen R, Schmitz RA 2017 sRNA154 a newly identified regulator of nitrogen fixation in *Methanosarcina mazei* strain Gö1. RNA Biol 14:1544–1558. doi:10.1080/15476286.2017.1306170. 28296572 PMC5785227

[24] Babski J, Haas KA, Näther-Schindler D, Pfeiffer F, Förstner KU, Hammelmann M, Hilker R, Becker A, Sharma CM, Marchfelder A, Soppa J 2016 Genome-wide identification of transcriptional start sites in the haloarchaeon *Haloferax volcanii* based on differential RNA-Seq (dRNA-Seq). BMC Genomics 17:629. doi:10.1186/s12864-016-2920-y. 27519343 PMC4983044

[25] Gebetsberger J, Zywicki M, Künzi A, Polacek N 2012 tRNA-derived fragments target the ribosome and function as regulatory non-coding RNA in *Haloferax volcanii*. Archaea 2012:260909. doi:10.1155/2012/260909. 23326205 PMC3544259

[26] Wurtzel O, Sapra R, Chen F, Zhu Y, Simmons BA, Sorek R 2010 A single-base resolution map of an archaeal transcriptome. Genome Res 20:133–141. doi:10.1101/gr.100396.109. 19884261 PMC2798825

[27] Jäger D, Sharma CM, Thomsen J, Ehlers C, Vogel J, Schmitz RA 2009 Deep sequencing analysis of the *Methanosarcina mazei* Go1 transcriptome in response to nitrogen availability. Proc Natl Acad Sci U S A 106:21878–21882. doi:10.1073/pnas.0909051106. 19996181 PMC2799843

[28] Li Y, Liu X, Huang L, Guo H, Wang XJ 2010 Potential coexistence of both bacterial and eukaryotic small RNA biogenesis and functional related protein homologs in Archaea. J Genet Genomics 37:493–503. doi:10.1016/S1673-8527(09)60069-2. 20816382

[29] Willkomm S, Oellig CA, Zander A, Restle T, Keegan R, Grohmann D, Schneider S 2017 Structural and mechanistic insights into an archaeal DNA-guided Argonaute protein. Nat Microbiol 2:17035. doi:10.1038/nmicrobiol.2017.35. 28319084

[30] Zander A, Willkomm S, Ofer S, van Wolferen M, Egert L, Buchmeier S, Stöckl S, Tinnefeld P, Schneider S, Klingl A, Albers S-V, Werner F, Grohmann D 2017 Guide-independent DNA cleavage by archaeal Argonaute from *Methanocaldococcus jannaschi*i. Nat Microbiol 2:17034. doi:10.1038/nmicrobiol.2017.34. 28319081 PMC7616673

[31] Willkomm S, Zander A, Gust A, Grohmann D 2015 A prokaryotic twist on Argonaute function. Life (Basel) 5:538–553. doi:10.3390/life5010538. 25692904 PMC4390867

[32] Jäger D, Pernitzsch SR, Richter AS, Backofen R, Sharma CM, Schmitz RA 2012 An archaeal sRNA targeting *cis*- and *trans*-encoded mRNAs via two distinct domains. Nucleic Acids Res 40:10964–10979. doi:10.1093/nar/gks847. 22965121 PMC3510493

[33] Bernick DL, Dennis PP, Lui LM, Lowe TM 2012 Diversity of antisense and other non-coding RNAs in archaea revealed by comparative small RNA sequencing in four *Pyrobaculum* species. Front Microbiol 3:231. doi:10.3389/fmicb.2012.00231. 22783241 PMC3388794

[34] Thomason MK, Storz G 2010 Bacterial antisense RNAs: how many are there and what are they doing? Annu Rev Genet 44:167–188. doi:10.1146/annurev-genet-102209-163523. 20707673 PMC3030471

[35] Conesa A, Madrigal P, Tarazona S, Gomez-Cabrero D, Cervera A, McPherson A, Szczeœniak MW, Gaffney DJ, Elo LL, Zhang X, Mortazavi A 2016 A survey of best practices for RNA-seq data analysis. Genome Biol 17:13. doi:10.1186/s13059-016-0881-8. 26813401 PMC4728800

[36] Li B, Dewey CN 2011 RSEM: accurate transcript quantification from RNA-Seq data with or without a reference genome. BMC Bioinformatics 12:323. doi:10.1186/1471-2105-12-323. 21816040 PMC3163565

[37] Li B, Ruotti V, Stewart RM, Thomson JA, Dewey CN 2010 RNA-Seq gene expression estimation with read mapping uncertainty. Bioinformatics 26:493–500. doi:10.1093/bioinformatics/btp692. 20022975 PMC2820677

[38] Wagner GP, Kin K, Lynch VJ 2012 Measurement of mRNA abundance using RNA-seq data: RPKM measure is inconsistent among samples. Theory Biosci 131:281–285. doi:10.1007/s12064-012-0162-3. 22872506

[39] Babski J, Maier LK, Heyer R, Jaschinski K, Prasse D, Jäger D, Randau L, Schmitz RA, Marchfelder A, Soppa J 2014 Small regulatory RNAs in Archaea. RNA Biol 11:484–493. doi:10.4161/rna.28452. 24755959 PMC4152357

[40] Storz G, Vogel J, Wassarman KM 2011 Regulation by small RNAs in bacteria: expanding frontiers. Mol Cell 43:880–891. doi:10.1016/j.molcel.2011.08.022. 21925377 PMC3176440

[41] He L, Hannon GJ 2004 MicroRNAs: small RNAs with a big role in gene regulation. Nat Rev Genet 5:522–531. doi:10.1038/nrg1379. 15211354

[42] De Lay N, Schu DJ, Gottesman S 2013 Bacterial small RNA-based negative regulation: Hfq and its accomplices. J Biol Chem 288:7996–8003. doi:10.1074/jbc.R112.441386. 23362267 PMC3605619

[43] Wheeler BS 2013 Small RNAs, big impact: small RNA pathways in transposon control and their effect on the host stress response. Chromosome Res 21:587–600. doi:10.1007/s10577-013-9394-4. 24254230

[44] Capy P, Gasperi G, Biemont C, Bazin C 2000 Stress and transposable elements: co-evolution or useful parasites? Heredity 85:101–106. doi:10.1046/j.1365-2540.2000.00751.x. 11012710

[45] Hoffmann L, Schummer A, Reimann J, Haurat MF, Wilson AJ, Beeby M, Warscheid B, Albers SV 2017 Expanding the archaellum regulatory network—the eukaryotic protein kinases ArnC and ArnD influence motility of Sulfolobus acidocaldarius. Microbiologyopen 6:e00414. doi:10.1002/mbo3.414. PMC5300886 27771939

[46] Albers S-V, Jarrell KF 2015 The archaellum: how Archaea swim. Front Microbiol 6:23. doi:10.3389/fmicb.2015.00023. 25699024 PMC4307647

[47] Mukhopadhyay B, Johnson EF, Wolfe RS 2000 A novel pH2 control on the expression of flagella in the hyperthermophilic strictly hydrogenotrophic methanarchaeaon *Methanococcus jannaschii*. Proc Nat Acad Sci U S A 97:11522–11527. doi:10.1073/pnas.97.21.11522. PMC17233 11027352

[48] Hendrickson EL, Liu Y, Rosas-Sandoval G, Porat I, Söll D, Whitman WB, Leigh JA 2008 Global responses of *Methanococcus maripaludis* to specific nutrient limitations and growth rate. J Bacteriol 190:2198–2205. doi:10.1128/JB.01805-07. 18203827 PMC2258866

[49] Xia Q, Wang T, Hendrickson EL, Lie TJ, Hackett M, Leigh JA 2009 Quantitative proteomics of nutrient limitation in the hydrogenotrophic methanogen *Methanococcus maripaludis*. BMC Microbiol 9:149. doi:10.1186/1471-2180-9-149. 19627604 PMC2723118

[50] Esquivel RN, Pohlschroder M 2014 A conserved type IV pilin signal peptide H-domain is critical for the post-translational regulation of flagella-dependent motility. Mol Microbiol 93:494–504. doi:10.1111/mmi.12673. 24945931

[51] Tripepi M, Imam S, Pohlschröder M 2010 *Haloferax volcanii* flagella are required for motility but are not involved in PibD-dependent surface adhesion. J Bacteriol 192:3093–3102. doi:10.1128/JB.00133-10. 20363933 PMC2901708

[52] Tripepi M, You J, Temel S, Önder Ö Brisson D, Pohlschröder M 2012 N-glycosylation of *Haloferax volcanii* flagellins requires known Agl proteins and is essential for biosynthesis of stable flagella. J Bacteriol 194:4876–4887. doi:10.1128/JB.00731-12. 22730124 PMC3430349

[53] Darnell CL, Schmid AK 2015 Systems biology approaches to defining transcription regulatory networks in halophilic archaea. Methods 86:102–114. doi:10.1016/j.ymeth.2015.04.034. 25976837

[54] Stroud A, Liddell S, Allers T 2012 Genetic and biochemical identification of a novel single-stranded DNA-binding complex in *Haloferax volcanii*. Front Microbiol 3:224. doi:10.3389/fmicb.2012.00224. 22719738 PMC3376784

[55] Skowyra A, MacNeill SA 2012 Identification of essential and non-essential single-stranded DNA-binding proteins in a model archaeal organism. Nucleic Acids Res 40:1077–1090. doi:10.1093/nar/gkr838. 21976728 PMC3273820

[56] DeVeaux LC, ller JA, Smith J, Petrisko J, Wells DP, DasSarma S 2007 Extremely radiation-resistant mutants of a halophilic archaeon with increased single-stranded DNA-binding protein (RPA) gene expression. Radiat Res 168:507–514. doi:10.1667/RR0935.1. 17903038

[57] McCready S, Muller JA, Boubriak I, Berquist BR, Ng WL, Dassarma S 2005 UV irradiation induces homologous recombination genes in the model archaeon, *Halobacterium sp*. NRC-1. Saline Systems 1:3. doi:10.1186/1746-1448-1-3. 16176594 PMC1224876

[58] Müller A, Langklotz S, Lupilova N, Kuhlmann K, Bandow JE, Leichert LIO 2014 Activation of RidA chaperone function by N-chlorination. Nat Commun 5:5804. doi:10.1038/ncomms6804. 25517874 PMC4284807

[59] Lambrecht JA, Flynn JM, Downs DM 2012 Conserved YjgF protein family deaminates reactive enamine/imine intermediates of pyridoxal 5′-phosphate (PLP)-dependent enzyme reactions. J Biol Chem 287:3454–3461. doi:10.1074/jbc.M111.304477. 22094463 PMC3270999

[60] Dahl J-U, Gray MJ, Jakob U 2015 Protein quality control under oxidative stress conditions. J Mol Biol 427:1549–1563. doi:10.1016/j.jmb.2015.02.014. 25698115 PMC4357566

[61] Stutz J, Ackermann R, Fast JD, Barrie L 2002 Atmospheric reactive chlorine and bromine at the Great Salt Lake, Utah. Geophys Res Let 29:18-1–18-4. doi:10.1029/2002GL014812.

[62] Nyström T 2005 Role of oxidative carbonylation in protein quality control and senescence. EMBO J 24:1311–1317. doi:10.1038/sj.emboj.7600599. 15775985 PMC1142534

[63] Suzuki YJ, Carini M, Butterfield DA 2010 Protein carbonylation. Antioxid Redox Signal 12:323–325. doi:10.1089/ars.2009.2887. 19743917 PMC2821144

[64] Scharf B, Engelhard M 1993 Halocyanin, an archaebacterial blue copper protein (type I) from *Natronobacterium pharaonis*. Biochemistry 32:12894–12900. doi:10.1021/bi00210a043. 8251512

[65] Lindas A-C, Bernander R 2013 The cell cycle of archaea. Nat Rev Microbiol 11:627–638. doi:10.1038/nrmicro3077. 23893102

[66] Waters LS, Storz G 2009 Regulatory RNAs in bacteria. Cell 136:615–628. doi:10.1016/j.cell.2009.01.043. 19239884 PMC3132550

[67] Mack GS 2007 MicroRNA gets down to business. Nat Biotechnol 25:631–638. doi:10.1038/nbt0607-631. 17557095

[68] Dyall-Smith ML 2001 The Halohandbook. Protocols for haloarchaeal genetics. http://www.haloarchaea.com/resources/halohandbook/.

[69] Zhang Z, Theurkauf WE, Weng Z, Zamore PD 2012 Strand-specific libraries for high throughput RNA sequencing (RNA-Seq) prepared without poly(A) selection. Silence 3:9. doi:10.1186/1758-907X-3-9. 23273270 PMC3552703

[70] Kim D, Langmead B, Salzberg SL 2015 HISAT: a fast spliced aligner with low memory requirements. Nat Methods 12:357–360. doi:10.1038/nmeth.3317. 25751142 PMC4655817

[71] Pertea M, Pertea GM, Antonescu CM, Chang T-C, Mendell JT, Salzberg SL 2015 StringTie enables improved reconstruction of a transcriptome from RNA-seq reads. Nat Biotech 33:290–295. doi:10.1038/nbt.3122. PMC4643835 25690850

[72] Pertea GM 2015 gffcompare. https://github.com/gpertea/gffcompare.

[73] Pertea M, Kim D, Pertea GM, Leek JT, Salzberg SL 2016 Transcript-level expression analysis of RNA-seq experiments with HISAT, StringTie and Ballgown. Nat Protoc 11:1650–1667. doi:10.1038/nprot.2016.095. 27560171 PMC5032908

[74] Liao Y, Smyth GK, Shi W 2014 featureCounts: an efficient general purpose program for assigning sequence reads to genomic features. Bioinformatics 30:923–930. doi:10.1093/bioinformatics/btt656. 24227677

[75] Love MI, Huber W, Anders S 2014 Moderated estimation of fold change and dispersion for RNA-seq data with DESeq2. Genome Biol 15:550. doi:10.1186/s13059-014-0550-8. 25516281 PMC4302049

[76] Robinson JT, Thorvaldsdottir H, Winckler W, Guttman M, Lander ES, Getz G, Mesirov JP 2011 Integrative genomics viewer. Nat Biotechnol 29:24–26. doi:10.1038/nbt.1754. 21221095 PMC3346182

[77] Altschul SF, Gish W, Miller W, Myers EW, Lipman DJ 1990 Basic local alignment search tool. J Mol Biol 215:403–410. doi:10.1016/S0022-2836(05)80360-2. 2231712

[78] Crooks GE, Hon G, Chandonia J-M, Brenner SE 2004 WebLogo: a sequence logo generator. Genome Res 14:1188–1190. doi:10.1101/gr.849004. 15173120 PMC419797

[79] Bailey TL, Boden M, Buske FA, Frith M, Grant CE, Clementi L, Ren J, Li WW, Noble WS 2009 MEME SUITE: tools for motif discovery and searching. Nucleic Acids Res 37:W202–W208. doi:10.1093/nar/gkp335. 19458158 PMC2703892

