# Supplementary material for: Transcriptional Landscape and Regulatory Roles of Small Noncoding RNAs in the Oxidative Stress Response of the Haloarchaeon Haloferax volcanii
Source: J Bacteriol. 2018 Apr 9;200(9):e00779-17. doi: 10.1128/JB.00779-17 (PMC5892119; doi:10.1128/JB.00779-17)
Supplement: Supplemental material [file supp_200_9_e00779-17__index.html]

Supplemental material 

# Transcriptional Landscape and Regulatory Roles of Small Noncoding RNAs in the Oxidative Stress Response of the Haloarchaeon Haloferax volcanii

## Supplemental material

- Supplemental file 1 -

  Fig. S1 (Cell survival)

  PDF, 2.3M
- Supplemental file 2 -

  Fig. S2 (Overlap lengths)

  PDF, 1.7M
- Supplemental file 3 -

  Fig. S3 (sRNAs contained a BRE and TATA box)

  PDF, 236K
- Supplemental file 4 -

  Fig. S4 (sRNA expression levels)

  PDF, 2.9M
- Supplemental file 5 -

  Fig. S5 (Expression versus transcript length)

  PDF, 6.1M
- Supplemental file 6 -

  Fig. S6 (sRNA expression)

  PDF, 3.2M
- Supplemental file 7 -

  Table S1 (sRNA expression)

  XLSX, 601K
- Supplemental file 8 -

  Table S2 (sRNA expression)

  XLSX, 111K
- Supplemental file 9 -

  Table S3 (sRNA expression)

  XLSX, 69K
- Supplemental file 10 -

  Table S4 (Gene expression)

  XLSX, 143K
