# Supplementary figures and images for: Transcriptional Landscape and Regulatory Roles of Small Noncoding RNAs in the Oxidative Stress Response of the Haloarchaeon Haloferax volcanii
Source: J Bacteriol. 2018 Apr 9;200(9):e00779-17. doi: 10.1128/JB.00779-17 (PMC5892119; doi:10.1128/JB.00779-17)

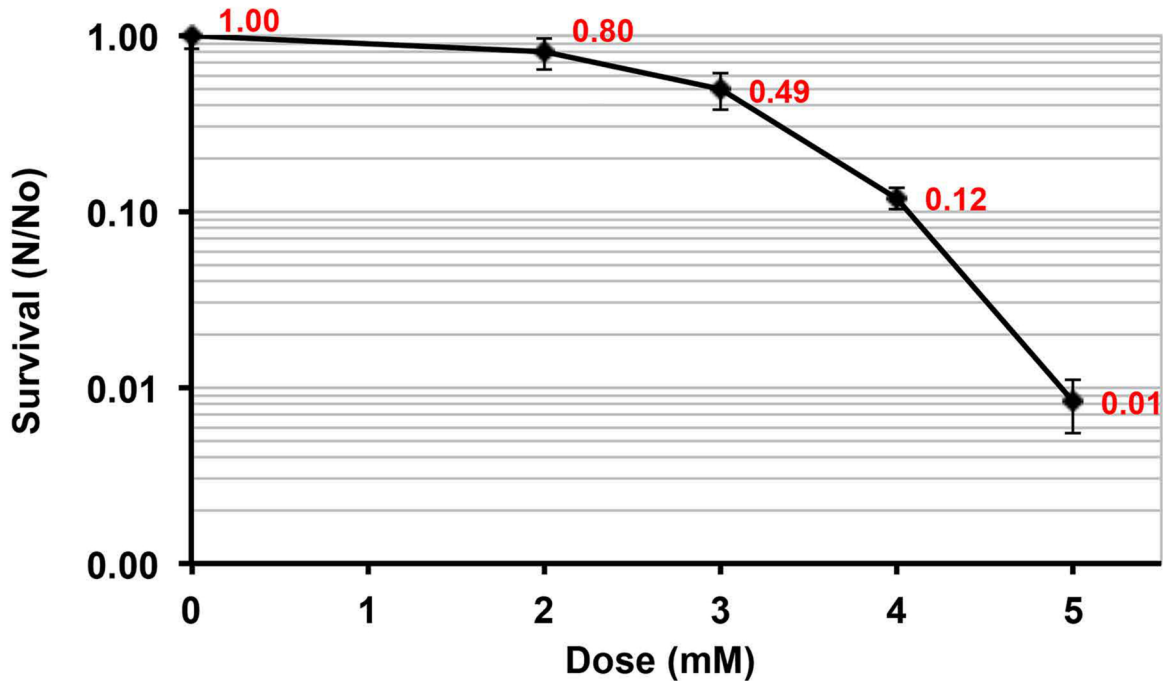

Supplement: Supplemental material [file JB.00779-17_zjb999094714s1.pdf]

**A**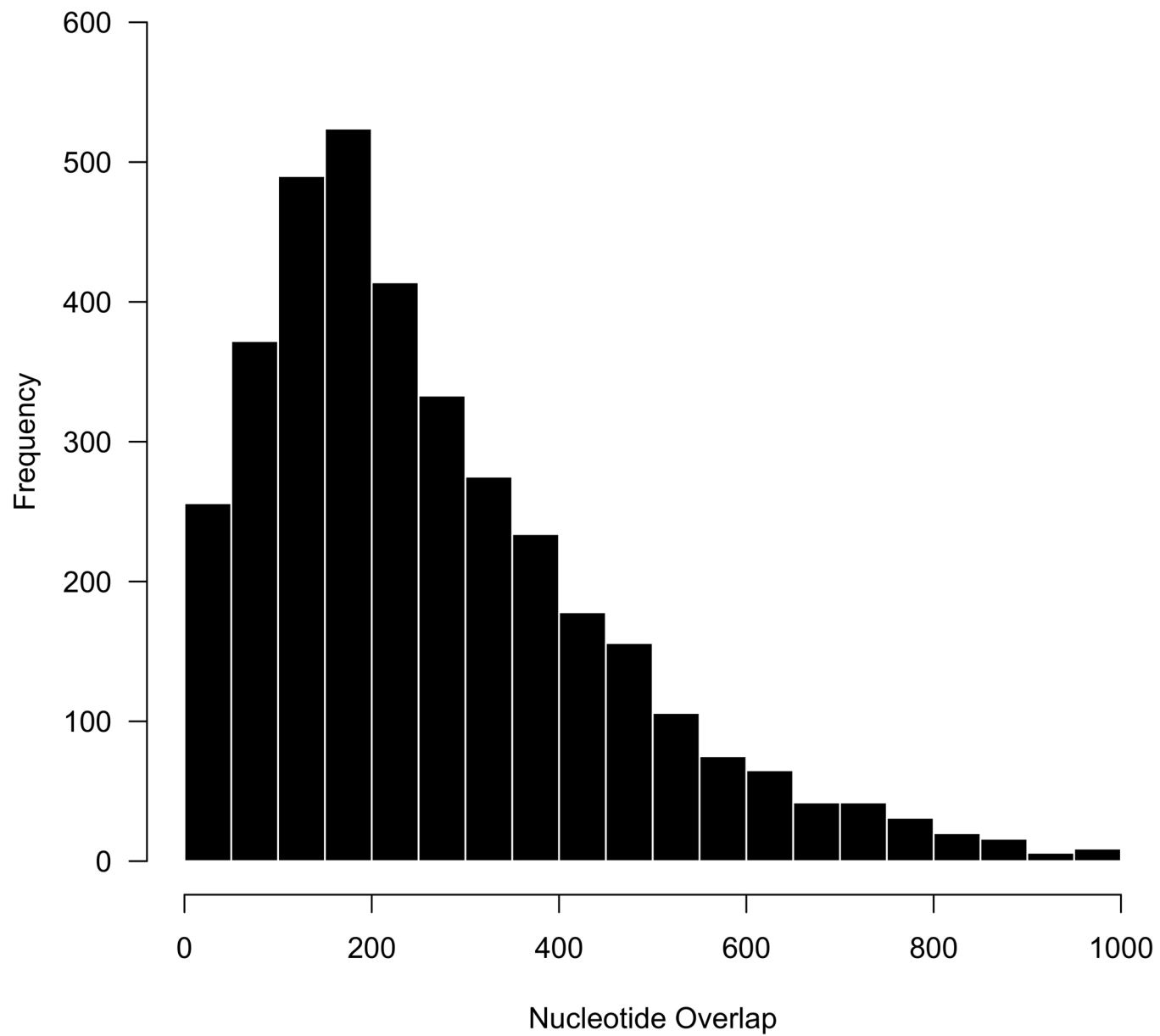**B**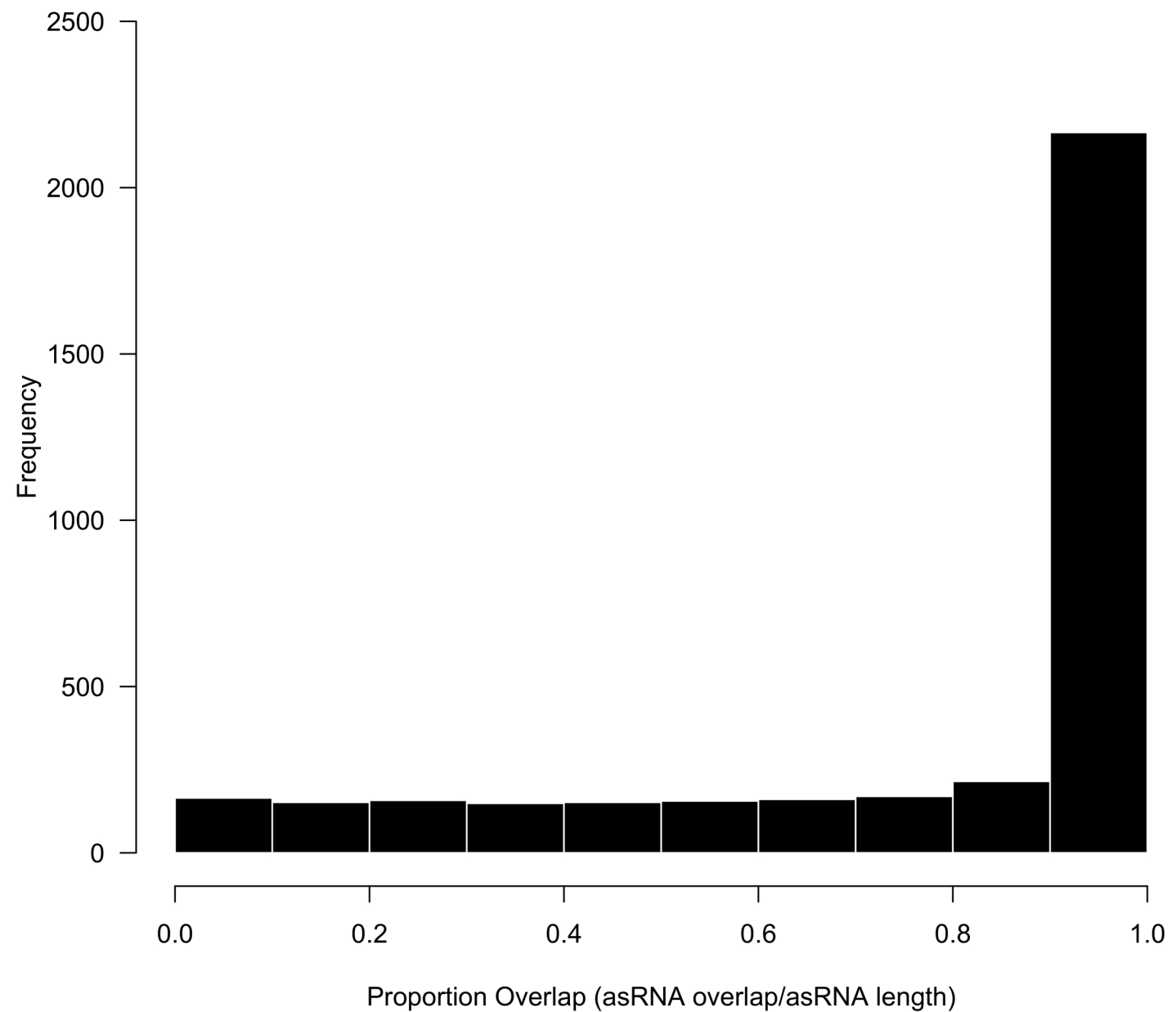

Supplement: Supplemental material [file JB.00779-17_zjb999094714s2.pdf]

A

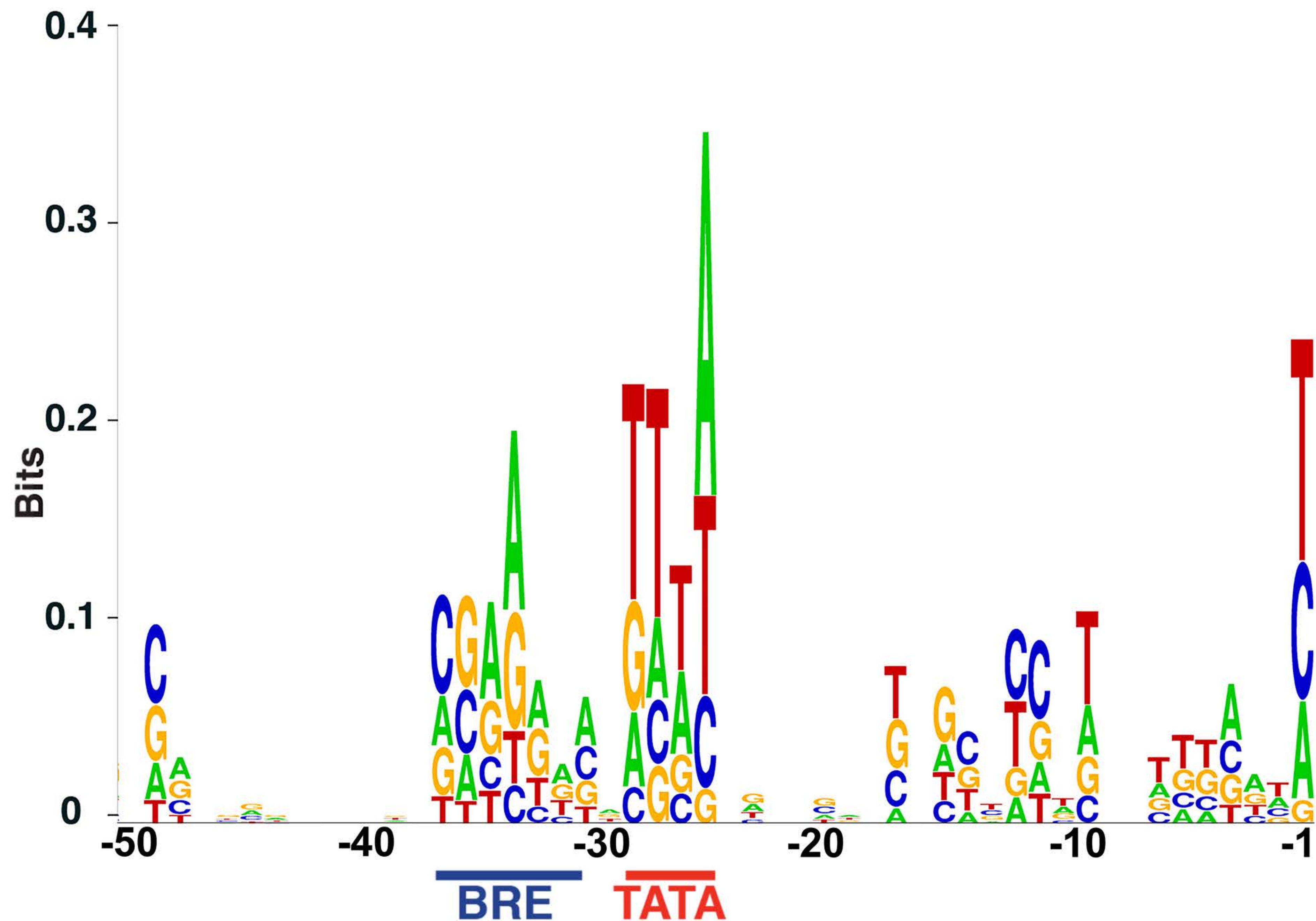

B

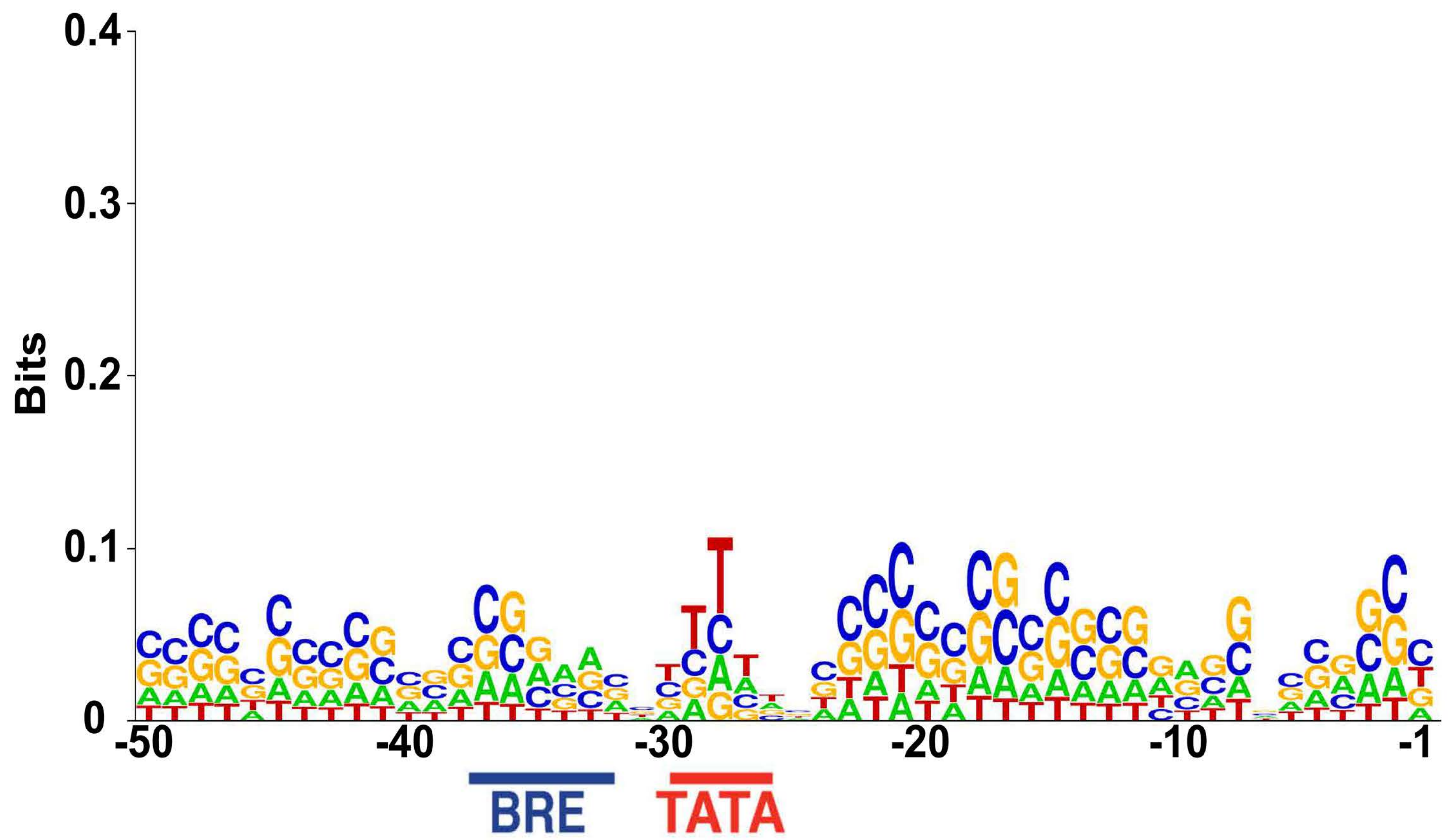

Supplement: Supplemental material [file JB.00779-17_zjb999094714s3.pdf]

**A**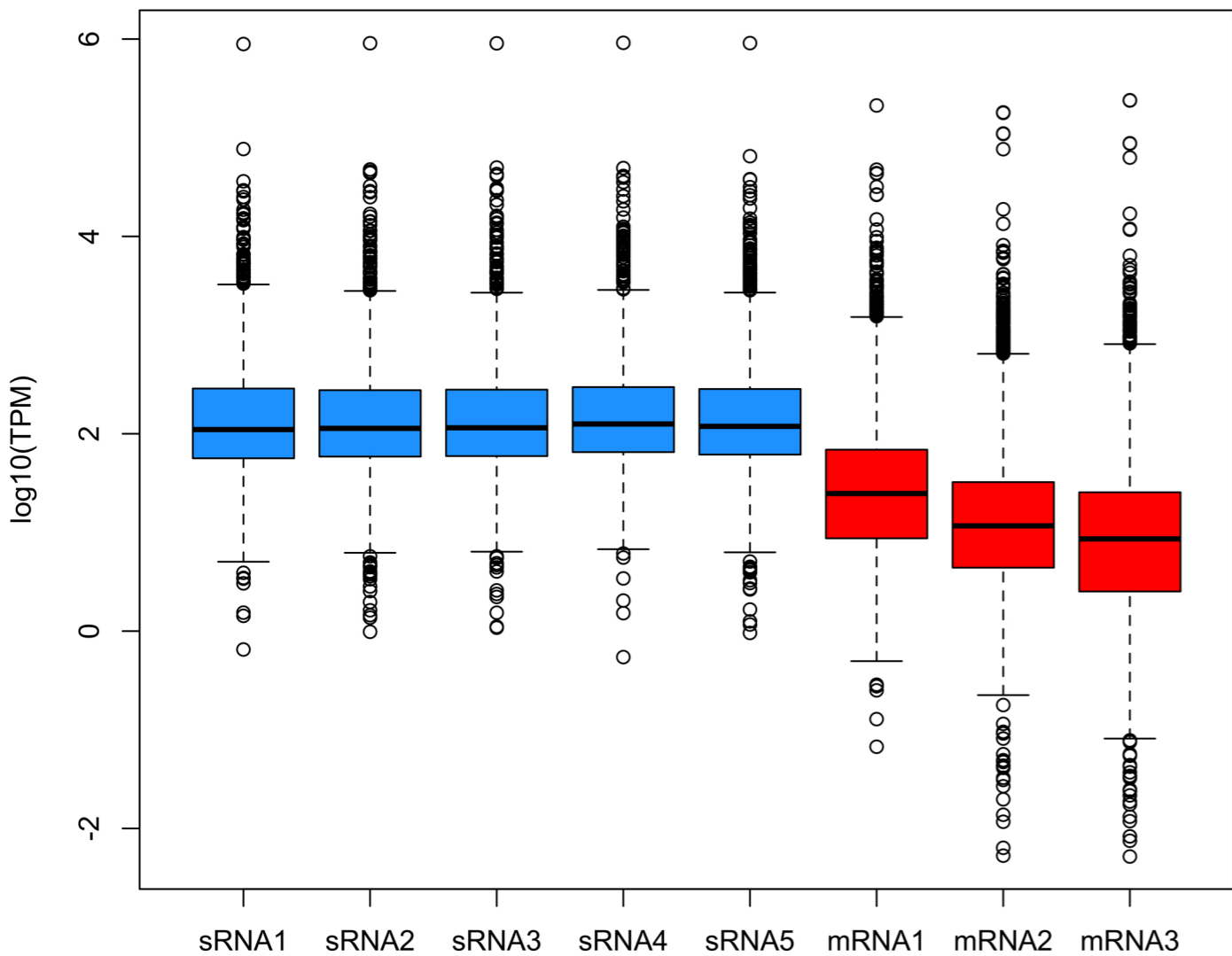**B**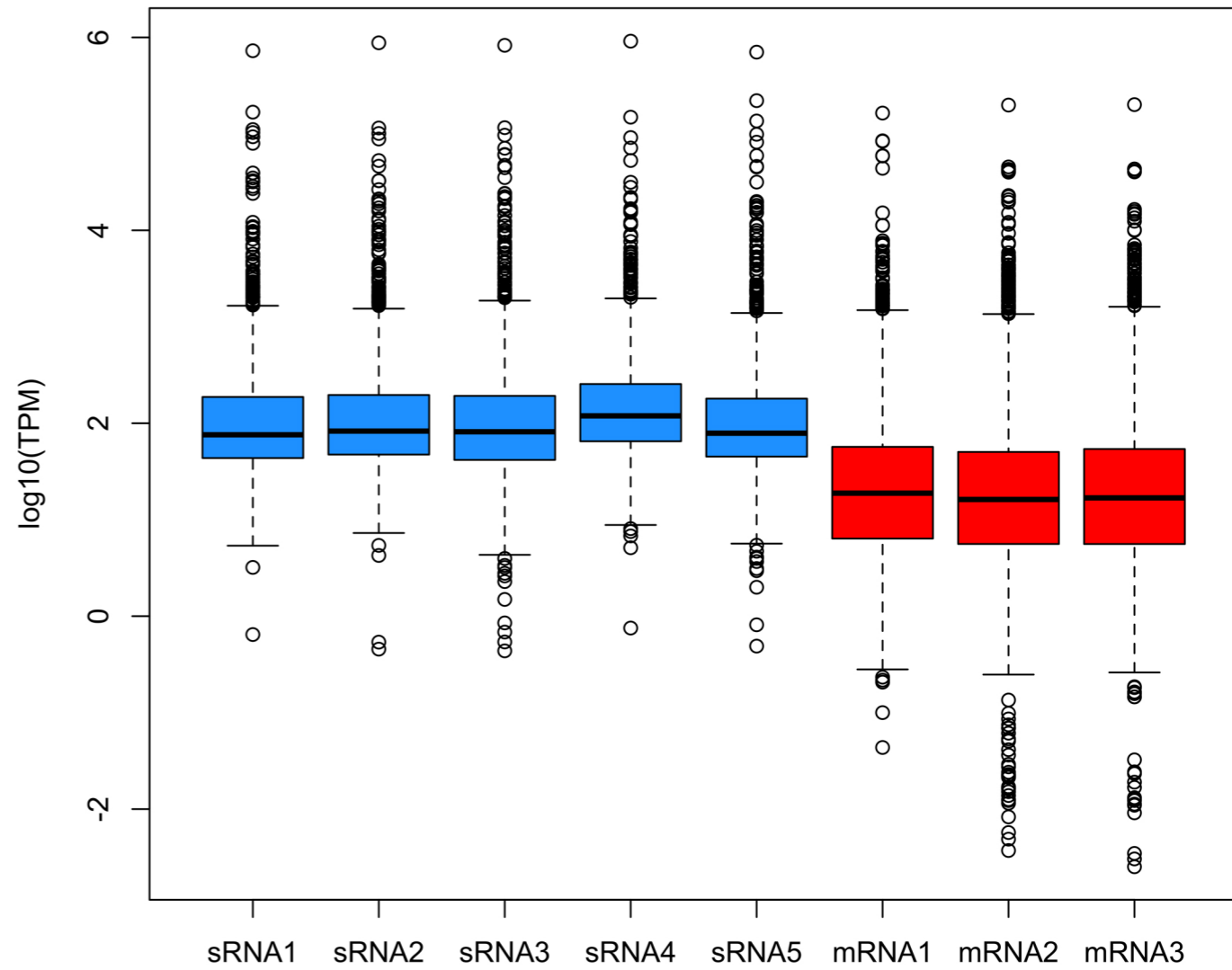

Supplement: Supplemental material [file JB.00779-17_zjb999094714s4.pdf]

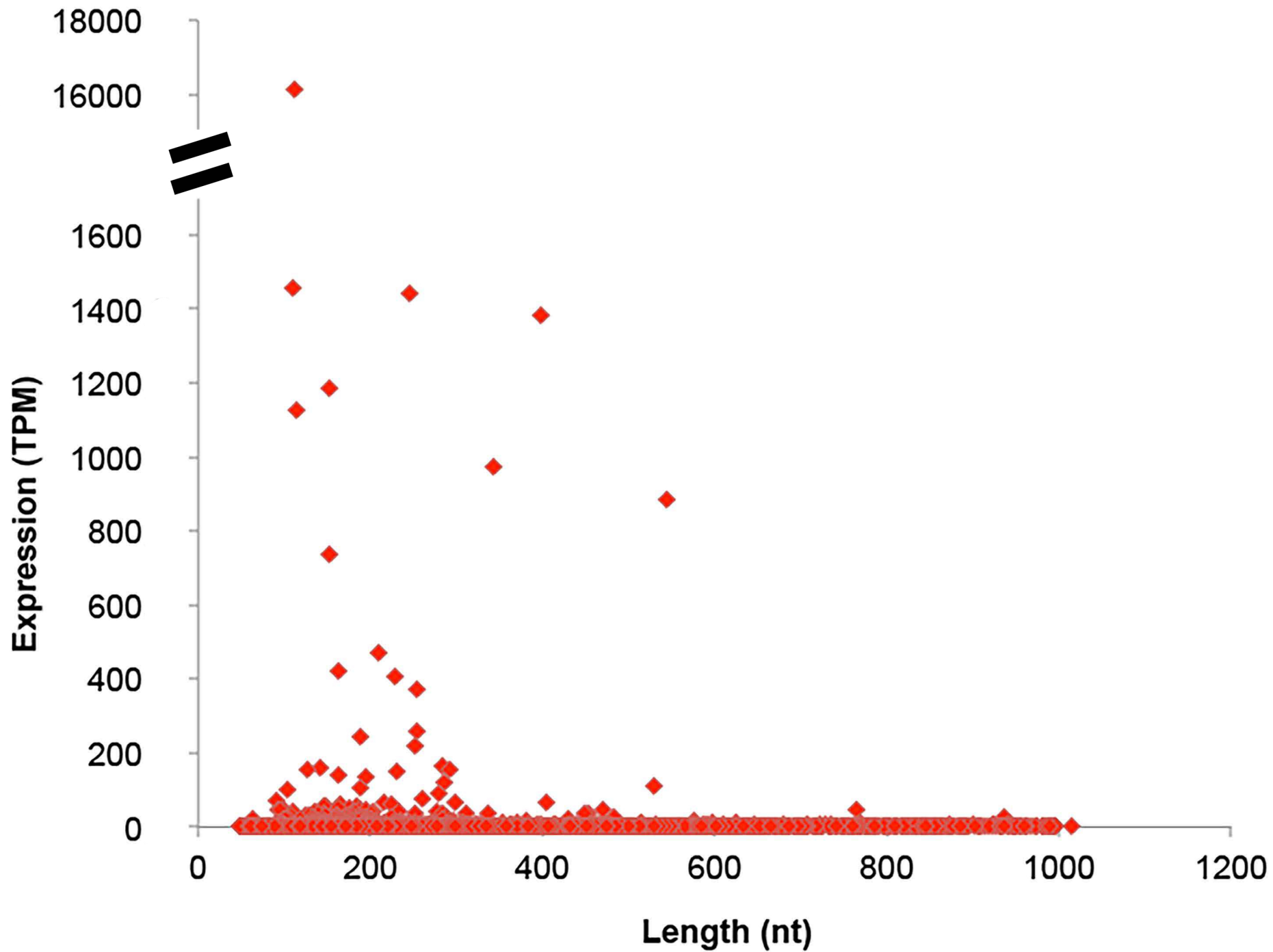

Supplement: Supplemental material [file JB.00779-17_zjb999094714s5.pdf]

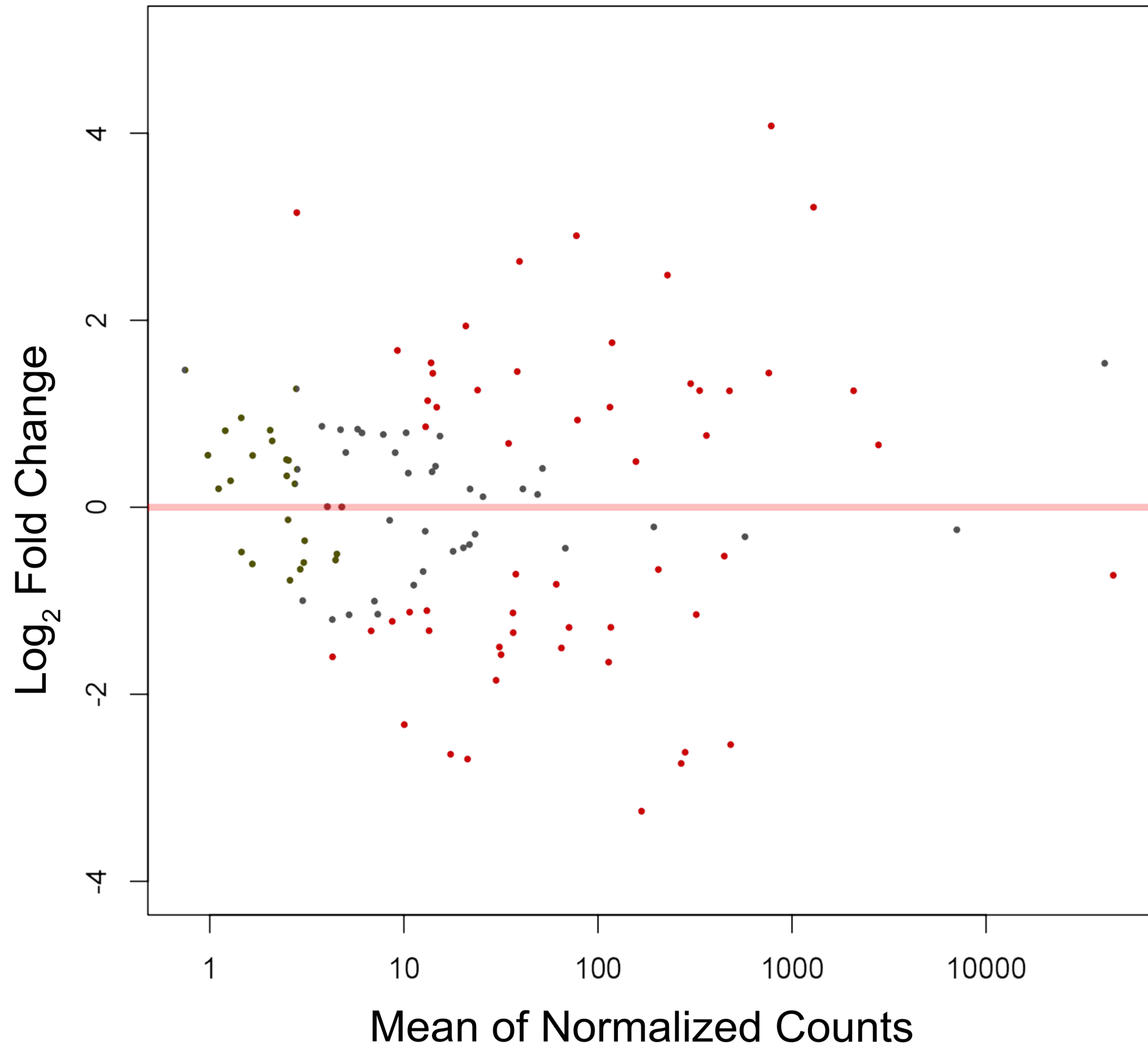

Supplement: Supplemental material [file JB.00779-17_zjb999094714s6.pdf]
